# Learned saccade readiness varies with fluctuations in sustained attention

**DOI:** 10.1038/s41598-025-14340-1

**Published:** 2025-08-23

**Authors:** Anthony W. Sali, Madison P. Shaver, Anna B. Toledo, Austin L. Torain, Isabel N. Flicker

**Affiliations:** Department of Psychology, Wake Forest University, 1834 Wake Forest Road, Winston-Salem, NC 27109 USA

**Keywords:** Cognitive neuroscience, Attention, Cognitive control, Human behaviour

## Abstract

Both the focus of sustained attention and an individual’s readiness to shift attention among spatial locations fluctuate over time. However, the interaction of these ongoing changes in attentional states remains unknown. In the current study, participants completed a modified gradual continuous performance task during which they monitored one of two lateralized streams of black and white images for the appearance of frequent target stimuli, withholding responses to foils. Periodically, a visual cue signaled participants to either maintain fixation at the current stream or to make a saccade to the opposing stream, and participants made a parity categorization for a digit appearing at the cued location. Trial-by-trial variation in pupil size, an indicator of arousal, accounted for both fluctuations in sustained attention and shift readiness but fluctuations in sustained attention were not associated with general modulations of shift readiness. Furthermore, we manipulated the frequency of gaze shift cues over time and observed that unexpected shift cues were most disruptive when participants lacked sustained focus, yielding a greater cost in saccade latencies than when the efficacy of sustained attention was high. Our results suggest that ongoing changes in sustained attention occur independently from gaze shifting readiness but carry consequences for learned saccade preparation.

**Supplementary Information:**

The online version contains supplementary material available at 10.1038/s41598-025-14340-1.

## Introduction

Attention is the process through which goal-relevant or physically salient stimuli receive preferential selection, known as attentional priority^1,2^. As behavioral goals change, individuals must suppress previously relevant stimuli, as well as the rules prioritizing their selection, and shift attentional selections. Consequently, the mechanisms responsible for shifts of attention among sensory representations have been an important area of research^3,4^. Both attention-shifting readiness, sometimes referred to as attentional flexibility, and the ability to focus attention on a single stimulus, referred to as sustained attention, vary over time^5–7^. However, the ways in which ongoing changes in sustained attention relate to those in shift readiness are unexplored.

To study fluctuations in sustained attention, researchers have relied on a combination of thought probes that require participants to report instances of mind-wandering^8^ as well as behavioral performance measures such as accuracy and response time (RT)^5,9^. Using a gradual onset version of the continuous performance task (CPT), Esterman and colleagues^10^ found that moment-by-moment variation in target detection RTs covaried with activity in the brain’s default mode network (DMN). An increase in DMN activity was associated with low RT variability, an indication of effective sustained attention. This finding has been replicated in several studies and distinguished from periods of self-reported mind-wandering^10–12^. These simultaneous changes in sustained attention and brain activity may stem from fluctuations in arousal resulting from locus-coeruleus (LC) -norepinephrine neuromodulation of fronto-parietal control regions, which in turn suppress the DMN^13,14^. For example, pupil size is known to covary with arousal and has been linked to fluctuations in sustained attention in gradual continuous performance tasks^15^. Interestingly, spontaneous periods of low shift readiness are also associated with elevated activity in the DMN, suggesting a potential link between fluctuating states of sustained attention and shift readiness^7^. 

A growing body of research has demonstrated that individuals track the likelihood of shifting attention to anticipate future demands and adjust shift readiness accordingly^16–18^. Although shifting attention typically incurs slower RTs and lower accuracy, referred to as a shift cost, the magnitude of this cost decreases with increasing shift likelihood, reflecting a learned adjustment in shift readiness as individuals increase their preparation to shift^16,17^. Changes in shift readiness are well accounted for by reinforcement learning models^19,20^, in which it is assumed that accurate, fast performance is intrinsically rewarding and sought after by participants, and mirror similar findings in the domain of task-switching and cognitive conflict control^4,21,22^.

Despite progress in characterizing the behavioral consequences and neural mechanisms associated with fluctuations in sustained attention and shift readiness, the extent to which these cognitive processes interact remains unknown. Understanding this potential interaction is important for several reasons. First, we will gain greater insight into the diversity or unity of attentional control operations by testing whether states of sustained attention and shift readiness are dissociable. Second, probing the interaction of sustained attention and shift readiness will provide a more nuanced explanation of moment-by-moment changes in behavior, reflecting the ways in which each control operation enhances or limits the efficacy of the other. Previous studies have suggested a distinction exists between internal forms of attention (selecting a task from working memory) and external forms of attention (selecting a spatial location in the visual field)^23^. However, sustained attention and shift readiness relate to both internal and external selection. Attention may be sustained on both external stimuli or internal representations, and learning influences shift readiness for both external spatial orienting^16,17^ and internal task-switching^22^. Therefore, sustained attention and shift readiness may be better thought of as factors that vary over time rather than across modalities, leaving the extent to which they interact unknown.

In the current study, we simultaneously tracked fluctuations in sustained attention and gaze shifting readiness, using a variant of the gradual CPT^5^. Participants periodically received visual cues to make a saccade (shift gaze) across two scene streams or hold attention while we manipulated the likelihood of cued saccades over time. On these embedded hold and shift gaze trials, participants made a parity categorization for a digit appearing at the cued location. Throughout, we refer to this measure of shift readiness as digit categorization RT. We additionally measured shift readiness with digit categorization accuracy and saccade latencies. As in previous studies^5^, we used ongoing changes in CPT RT variability as a measure of sustained attention efficacy. Thus, unlike previous studies of attentional flexibility^7,16–18^, we were able to track fluctuating states of attentional selection during the moments between cued shifts and holds. We first tested the relationships among arousal, sustained attention, and readiness to shift attention by generating a saccade. Consistent with arousal models of attentional fluctuations, we hypothesized that both CPT RT variability and digit categorization RT costs would vary with pupil size according to a U-shaped pattern with the lowest CPT RT variability and the largest digit categorization RT shift costs associated with intermediate arousal levels^15^.

Next, we tested whether the efficacy of sustained attention minimizes or magnifies the extent to which individuals adjust shift readiness based on changing shift likelihoods. As in previous studies^16–18^, we hypothesized that the magnitude of digit categorization RT and accuracy shift costs would be larger in low shift likelihood blocks than in high shift likelihood blocks, referred to below as a statistical learning effect. We tested whether the magnitude of the statistical learning effect varied in relation to fluctuating states of sustained attention. One possible way that sustained attention may influence shift readiness is through a filtering of information at the stage of encoding. For example, if sustained attention is required for the encoding of statistical likelihoods^24^, we would find reduced statistical learning effects during periods of high CPT RT variability (low efficacy sustained attention). However, it is also possible that high efficacy sustained attention (marked by periods with low CPT RT variability) could play a shielding role, insulating orienting processes from costs associated with violations of expectations. In this case, we would find larger statistical learning effects in periods of poor spatial attention since high efficacy sustained attention would better prepare individuals to overcome the presentation of an unexpected stimulus (e.g., receiving a hold cue in a high shift likelihood block).

Finally, it is also possible that the efficacy of sustained attention is at least partially yoked to ongoing changes in shift readiness or to the validity of shift likelihood predictions, reflecting a partial dependence of the two operations. Under this dependence hypothesis, trial-by-trial CPT RTs would vary less in low shift readiness blocks relative to high shift readiness blocks. Relatedly, the presentation of an unexpected cue stimulus may disrupt sustained attention, making post-cue CPT RTs deviate from an individual’s mean CPT RT to a greater extent following a violation of expectations than following receipt of a high likelihood cue stimulus. Conversely, an account of independence would predict that, regardless of whether shift readiness and sustained attention both vary with arousal, there is no shared variability between the two.

## Results

**Fig. 1:**
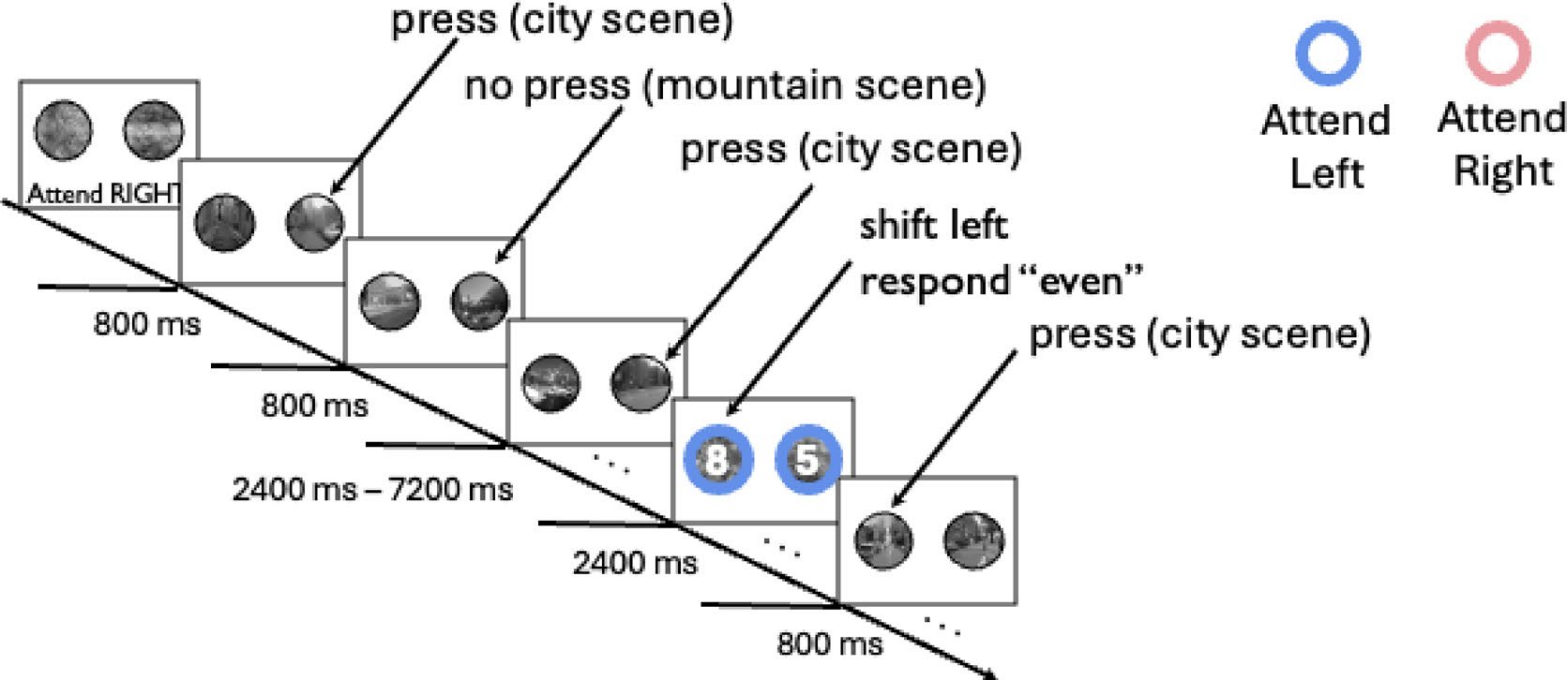
Gaze shifting gradual continuous performance task. Participants monitored a stream of gradually updating city and mountain images, pressing a button for each city detected. The appearance of colored borders indicated where participants should next focus their gaze with blue signaling left and pink signaling right. After directing their attention according to the color cue, participants made a parity discrimination of the digit at the cued location.

Participants completed a gaze shifting variant of the gradual CPT that combined a measure of moment-by-moment fluctuations in states of sustained attention with a measure of learned gaze-shifting readiness. The stimuli consisted of black and white city and mountain scenes^5^ positioned in circular apertures on the left and right sides of the display (see Fig. 1). Participants were instructed to attend to only one stream at a time and the scenes in both streams gradually changed in coherence such that one image slowly appeared while the other disappeared over the course of approximately 800 ms using a linear pixel-by-pixel interpolation. Participants made a manual response upon detecting each city scene at the attended location, which we used to derive a moment-by-moment measure of the efficacy of sustained attention. As in previous studies, we refer to this measure of CPT RT variability as the variance time course (VTC; see Methods). Participants were to withhold their response when the to-be-attended image was a rare mountain scene.

We embedded periodic attentional orienting trials, which required participants to either make a saccade or maintain fixation at the attended location, among the continuous performance trials described above. At the start of these orienting trials, identical colored borders appeared at each stream and the scenes were replaced by phase-scrambled images that continued to gradually change at the same rate. The images in both streams immediately preceding the colored border onset were also scrambled to avoid overlap of the continuous performance and orienting tasks. This meant that the onset of the colored borders was separated from the 100% coherence point of the preceding city or mountain scenes by approximately 800 ms. Participants were to focus their gaze on the left stream if the borders were blue and the right stream if the borders were pink, thereby requiring a saccade if the previously fixated location did not match the newly cued location. Two randomly generated digits appeared superimposed on top of the scrambled scenes at the same time as the colored border onset and participants made a speeded button press to indicate the parity of the digit at the cued location, yielding digit categorization RTs. After an approximately 2400 ms response window, the borders offset, and participants returned to detecting city scenes among mountains in the newly attended stream. If the participant made a fixation on the to-be-ignored side of the display prior to the cue onset, the digits were replaced with two Xs and the trial was designated as a response error. We divided each run of the task into four equal length blocks with two blocks per run containing a high likelihood of spatial attention shifting and two blocks per run with a low likelihood of spatial attention shifting to test the interaction of learned shift readiness with sustained attention.

Here, we focus on three interrelated questions. First, we used linear mixed effects models to test the relationships among arousal, CPT RT variability, and ongoing changes in shift readiness as measured by digit categorization RTs and saccade latencies. We selected pupil size as an indicator of moment-by-moment arousal^25^. Second, we tested whether the degree to which shift readiness varies according to previous learning changed based on sustained attention states. We classified epochs of the task as *in the zone* (low CPT RT deviance from an individual’s mean) and *out of the zone* (high CPT RT deviance from their mean) and tested whether the increase in shift costs and saccade latencies associated with a low shift likelihood varied based on this classification. Finally, we tested whether ongoing changes in shift likelihood influence states of sustained attention by comparing the standard deviation of CPT RTs falling in high and low shift likelihood blocks and by testing whether CPT RTs were more likely to deviate from each participant’s mean following unexpected cue outcomes.

### Linking arousal, sustained attention, and shift readiness

**Fig. 2:**
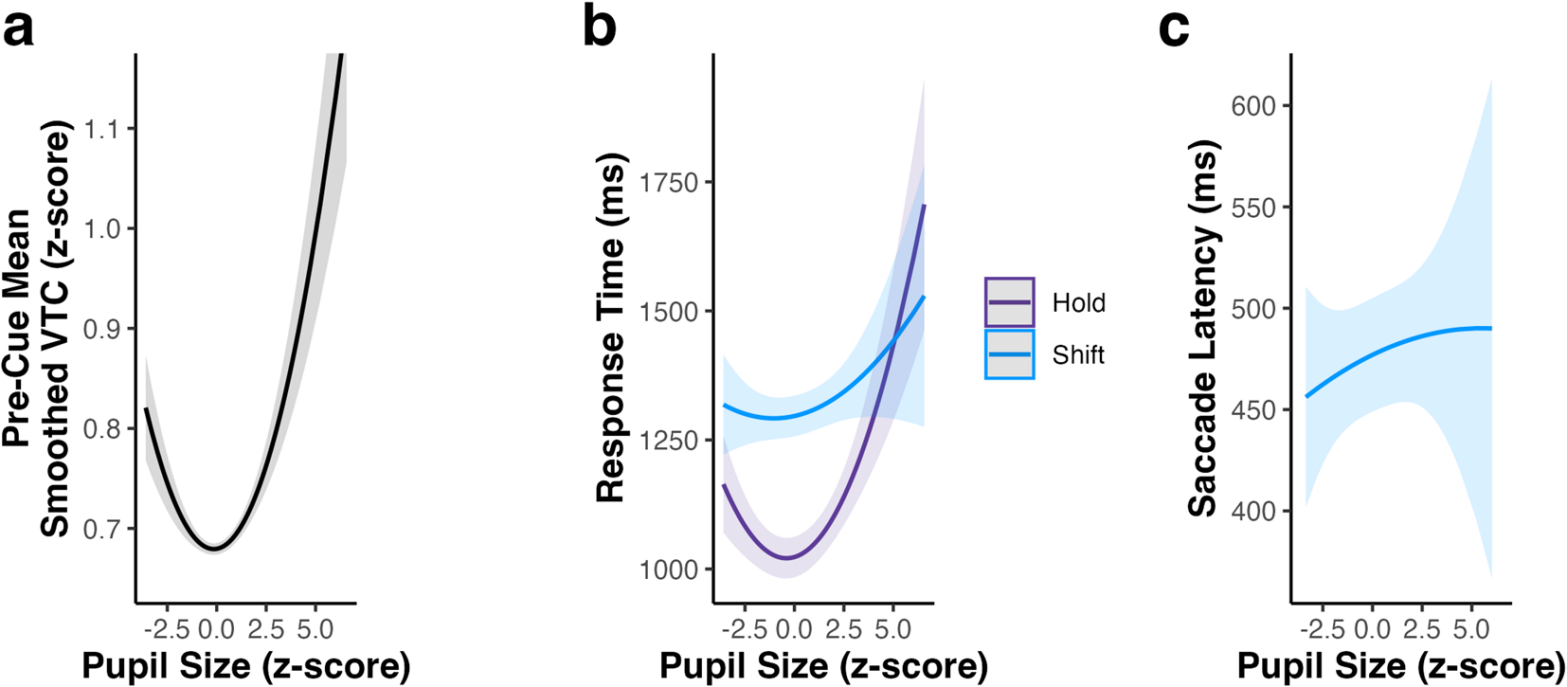
Marginal estimated fixed effects for (**a**) pre-cue RT, (**b**) digit categorization response time following a hold or shift cue, and (**c**) saccade latency as a function of pretrial pupil size. Shaded regions denote 95% confidence intervals.

To better understand whether fluctuations in sustained attention and shift readiness in part covary according to the same mechanism, we tested the relationships with the pupil size preceding the attention cue, referred to below as the pre-cue size (see Fig. 2), which has been shown to vary with states of arousal^13,15^. We averaged smoothed VTC values within a pre-cue temporal window, defined as the values associated with the two CPT trials and the scrambled image (approximately 2400 ms with respect to the beginning of each image transition) prior to each attention cue onset. The smoothing meant that the estimate of sustained attention reflected CPT RTs falling both before and after the current cue and are therefore not a measure of preparatory attentional states alone. Pre-cue pupil size was defined as the average size for the 500 ms preceding each attention cue. Here, and in all analyses of the shift readiness data below, we only analyzed trials in which the participant made an accurate digit categorization manual response and met a series of gaze position criteria (see Methods), unless stated otherwise. Furthermore, for all pupil size analyses, we excluded any trials (0.21% of all trials) where there was otherwise valid pupil size data (see Methods) but the participant did not maintain fixation at the accurate location during the 500 ms pre-cue window defined above. First, we tested the relationship between pretrial pupil size and sustained attention with a linear mixed-effects model with linear and quadratic fixed effects of pupil size to capture different patterns of influence. The model also accounted for individual differences by including random slopes for the linear and quadratic relationships, in addition to random intercepts, to account for baseline differences in sustained attention across participants (see Fig. 2A; Supplemental Table S1). In support of previous studies^15^, we observed a significant quadratic relationship with the lowest VTC values at intermediate pupil sizes and higher VTC values (poorer sustained attention) as pupil size deviated, *b* = 0.012, *SE* = 0.002, *t*(108.59) = 6.57, *p* < 0.001, ${R}_{p}^{2}$ = 0.006. There was no significant linear effect, *b* = 0.003, *SE* = 0.003, *t*(80.30) = 1.06, *p* = 0.294, ${R}_{p}^{2}$ < 0.001 and the marginal *R*^*2*^ was 0.007.

Given the quadratic relationship between pupil size and sustained attention, we next tested whether digit categorization RTs and saccade latencies on attentional orienting trials also varied according to pretrial pupil size. RTs and saccade latencies were measured as the length of time between the onset of the orienting cue and the participant’s manual response or initiation of a saccade between the two streams. As illustrated in Fig. 2B, we tested the relationship between pre-cue pupil size and digit categorization RTs with a linear mixed-effects model containing fixed effects of cue type (shift vs. hold), linear and quadratic pupil size, and the interactions between cue type and pupil size effects (marginal *R*^*2*^ = 0.122). The random effects structure included random slopes for cue type, the linear and quadratic effects of pupil size, the interaction between cue type and the linear pupil size effect, and random intercepts. Participants were slower on shift trials than on hold trials, *b* = -136.60, *SE* = 6.26, *t*(90.91) =  -21.82, *p* < 0.001, ${R}_{p}^{2}$ = 0.095, and there were linear, *b* = 9.94, *SE* = 4.19, *t*(78.51) = 2.37, *p* = 0.020, ${R}_{p}^{2}$ = 0.001, and quadratic, *b* = 9.10, *SE* = 2.30, *t*(66.51) = 3.95, *p* < 0.001, ${R}_{p}^{2}$ = 0.002, effects of pupil size on digit categorization RT (see Supplemental Table S2). However, these effects were qualified by a significant interaction of cue type and the quadratic term, *b* = 5.01, *SE* = 1.92, *t*(2,422.86) = 2.61, *p* = 0.009, ${R}_{p}^{2}$ = 0.001, as digit categorization RTs for hold gaze trials varied according to a U-shape more strongly than did shift trials. The linear effect did not vary according to cue type, *b* = 1.38, *SE* = 3.54, *t*(79.21) = 0.39, *p* = 0.698, ${R}_{p}^{2}$ < 0.001. To test the relationship between pretrial pupil size and saccade latencies for each type of attentional orienting trial, we used another linear mixed-effects model of saccade latencies with fixed effects of linear and quadratic pupil size, random slopes for the linear and quadratic effects, and random intercepts (marginal *R*^*2*^ < 0.001; see Fig. 2C). There were no significant linear, *b* = 4.76, *SE* = 3.35, *t*(79.43) = 1.42, *p* = 0.158, ${R}_{p}^{2}$ < 0.001, or quadratic, *b* = -0.43, *SE* = 1.80, *t*(26.44) = -0.24, *p* = 0.812, ${R}_{p}^{2}$ < 0.001, relationships between pretrial pupil size and saccade latency (see Supplemental Table S3). Taken together, there was a larger difference between shift and hold trial digit categorization RTs (the shift cost) when participants had an intermediate level of arousal, as measured by pupil size, than at the extremes, suggesting that moderate levels of arousal promote both stable states of sustained attention and low shift readiness.

### The interaction of sustained attention and learned shift readiness

**Fig. 3:**
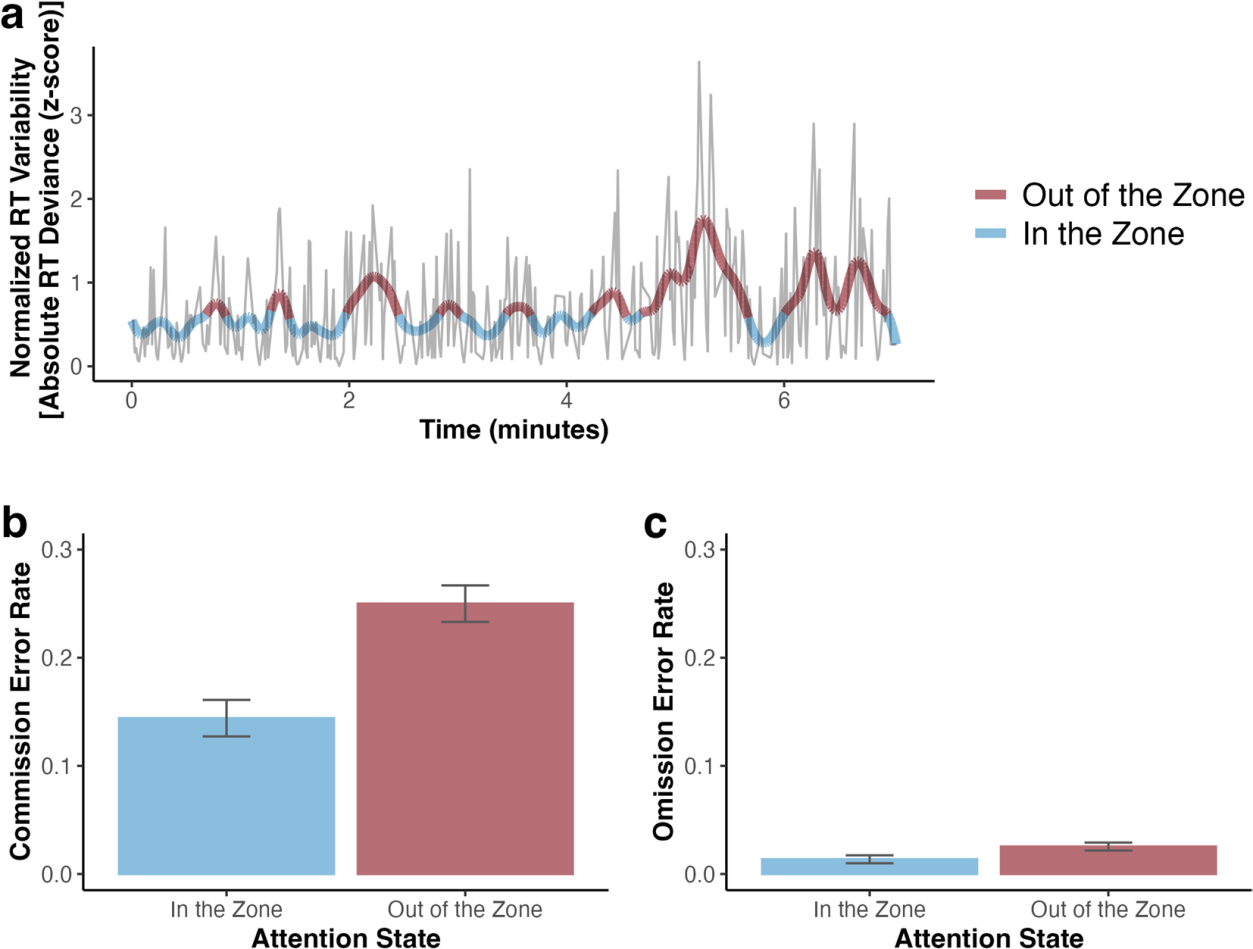
Definition of *in the zone* and *out of the zone* epochs. (**a**) Example VTC from a single run of one participant. Raw RT variability is plotted in light gray. The thick line represents the smoothed time course with the run divided into *in the zone* and *out of the zone* epochs according to a median split. (**b**) Commission and (**c**) omission error rates as a function of sustained attention state. Error bars denote difference and correlation-corrected within-subject 95% confidence intervals^27^. Correlation adjustment was carried out using the Cousineau-Morey approach^28,29^.

Next, we tested whether the impact of trial history on moment-by-moment shift readiness, a reflection of previous learning, varied according to fluctuating states of sustained attention. In particular, this analysis tested whether the difference in shift costs attributable to shift likelihood would vary based on CPT RT variability. As illustrated in Fig. 3A, we divided epochs as *in the zone* and *out of the zone* according to a median split of smoothed VTC values on a run-by-run basis. While the VTC provides a continuous measure of sustained attention, we elected to define a binary classification of high (*in the zone*) and low (*out of the zone*) efficacy sustained attention, as in previous studies^5,11,26^, to further reduce noise in our definition of sustained attention states. To verify that this division effectively characterized fluctuations in performance, we tested whether differences in commission and omission error rates for the city detection task varied across these periods. As expected, individuals made significantly more commission, *t*(75) = 12.49, *p*  < 0.001, *d*_*z*_ = 1.433, and more omission, *t*(75) = 6.37, *p* < 0.001, *d*_*z*_ = 0.731 errors when *out of the zone* than when *in the zone*, suggesting that our VTC measure accounted for fluctuations in sustained attention (see Fig. 3B,C).

#### Digit categorization markers of shift readiness

Given the delineation of trials into *in the zone* and *out of the zone* epochs described above, we tested whether shift readiness varied according to states of sustained attention and statistical learning. We labeled whether the participant was *in the zone* or *out of the zone* for each attentional orienting trial by averaging the smoothed VTC values assigned to the VTC pre-cue window defined above and comparing the average to the run’s median VTC score. Due to this data-driven approach, we could not control the number of high and low likelihood trials falling into each cell of the design. This resulted in few occurrences of some trial types for some participants and one participant who had no trials in one cell of the design at all. When excluding this individual, the number of trials per condition for each participant ranged from 3 to 43, potentially contributing to noisier estimates in some conditions than in others. We therefore report both repeated measures ANOVAs with the participant who had missing data excluded as well as a linear mixed-effects model, which retains the participant with missing data.

**Fig. 4:**
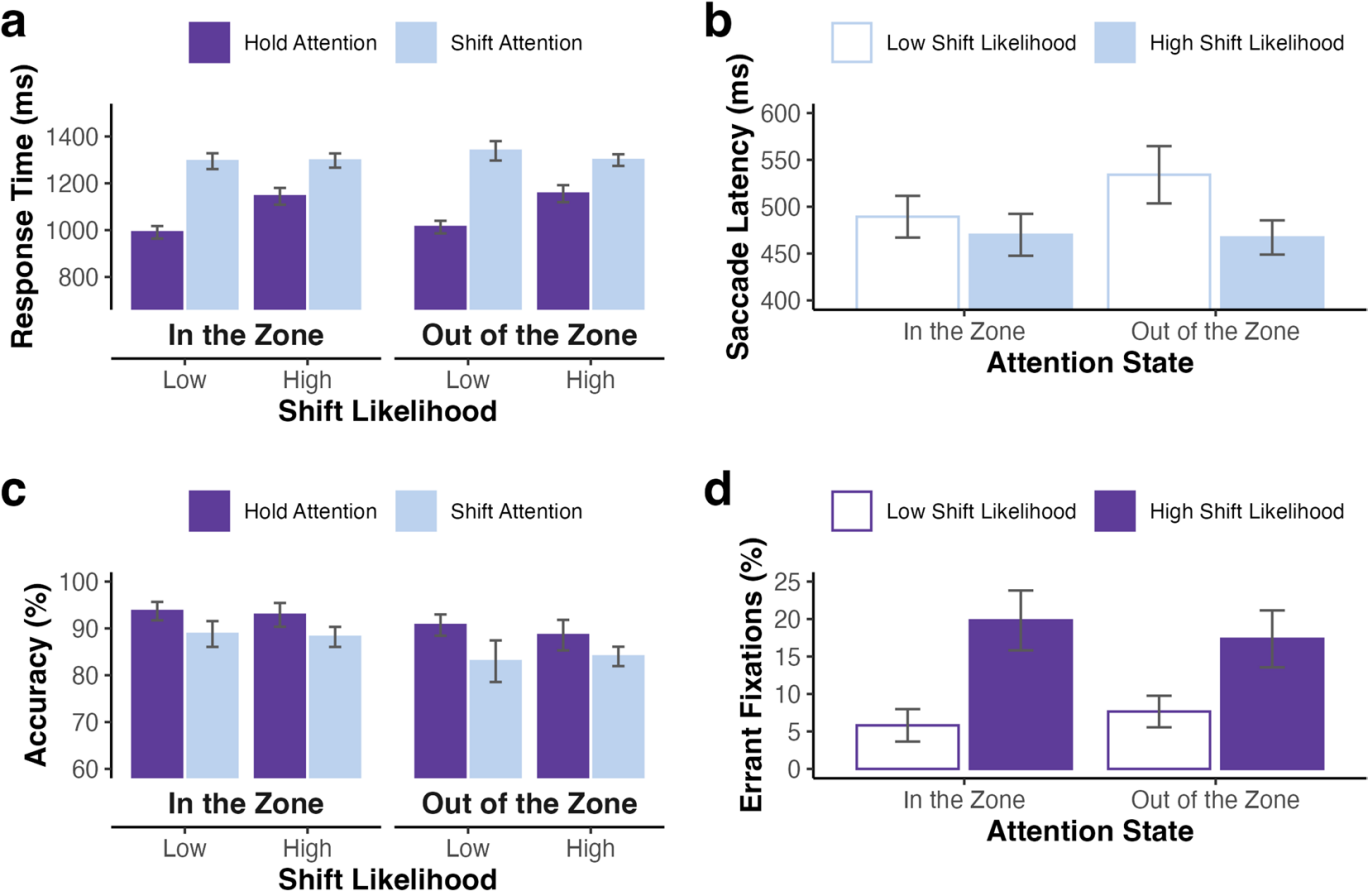
The interaction of cue type, shift likelihood, and sustained attention state. (**a**) Digit categorization RTs and (**b**) saccade latencies as a function of trial type, shift likelihood context, and sustained attention state. A single participant is excluded from A-B due to missing data. (**c**) Combined behavioral accuracies for manual responses and gaze position as a function of trial type, shift likelihood, and sustained attention state. (**d**) The percentage of fixations made at the to-be-ignored location prior to manual response varied according to shift likelihood. Error bars denote difference and correlation-corrected within-subject 95% confidence intervals^27^. Correlation adjustment was carried out using the Cousineau-Morey approach^28,29^.

To investigate the interaction between sustained attention and learned shift readiness, we conducted a repeated measures ANOVA of digit categorization RTs, with factors of cue type (shift vs. hold), shift likelihood (low vs. high), and sustained attention state (*in the zone* vs. *out of the zone*). As illustrated in Fig. 4A, participants were slower on shift gaze trials than on hold gaze trials, *F*(1,74) =  393.08, *p* < 0.001, ${\widehat{\eta }}_{p}^{2}$ = 0.842, and in blocks with a high shift likelihood than those with a low shift likelihood, *F*(1,74) = 53.73, *p* < 0.001, ${\widehat{\eta }}_{p}^{2}$ = 0.421. The cost in digit categorization RT associated with shifting was significantly smaller in high shift likelihood blocks than in low shift likelihood blocks, *F*(1,74) =  146.20, *p* < 0.001, ${\widehat{\eta }}_{p}^{2}$ = 0.664, indicating learned adjustments in shift readiness. In addition to this evidence of learned shift readiness, we also observed a general cost associated with *out of the zone* periods such that participants were slower to respond than when *in the zone*, *F*(1,74) =  6.20, *p* = 0.015, ${\widehat{\eta }}_{p}^{2}$ = 0.077, regardless of cue type and shift likelihood. However, learned shift readiness did not differ for *in the zone* and *out of the zone* epochs, as indicated by the lack of a three-way interaction, *F*(1,74) =  1.37, *p* = 0.246, ${\widehat{\eta }}_{p}^{2}$ = 0.018. No other interactions reached significance, *F*s < 3.15, *p*s > 0.080.

To test the robustness of our findings from the ANOVA, we compared these results to those from a linear mixed-effects model with fixed effect regressors for the main effects and interactions of trial type (shift vs. hold), shift likelihood (low vs. high), and state (*in the zone* vs. *out of the zone*), as well as random slopes for each main effect and random intercepts (see Supplemental Table S4). As in the ANOVA, there were significant main effects of trial type, *b* = -113.74, *SE* = 5.98, *t*(83.71) = -19.03, *p* < 0.001, ${R}_{p}^{2}$ = 0.069, shift likelihood, *b* = 35.73, *SE* = 4.18, *t*(82.75) = 8.54, *p* < 0.001, ${R}_{p}^{2}$ = 0.007, and sustained attention state, *b* = -9.82, *SE* = 4.03, *t*(111.81) = -2.44, *p* = 0.016, ${R}_{p}^{2}$ = 0.001. A significant interaction of trial type by shift likelihood, *b* = 41.09, *SE* = 3.32, *t*(11,977.91) = 12.39, *p* < 0.001, ${R}_{p}^{2}$ = 0.010, again provided evidence of learned shift readiness. However, learned shift readiness did not differ for *in the zone* and *out of the zone* epochs, as indicated by the lack of a three-way interaction, *b* = -2.50, *SE* = 3.34, *t*(12,004.23) = -0.75, *p* = 0.454, ${R}_{p}^{2}$ < 0.001, reflecting no change in shift readiness with fluctuations in sustained attention. The remaining interactions also failed to reach significance, *t*s < 1.88, *p*s >  0.060 and the marginal *R*^*2*^ was 0.135.

#### Gaze markers of shift readiness

We next conducted the same analyses on saccade latencies to test the role of learning across differing states of sustained attention. While there was no significant three-way interaction in the RT analysis, saccade latencies provide a potentially more direct measure of attentional reorienting. Therefore, this analysis would shed additional light on whether trial history influenced moment-by-moment shift readiness to differing extents dependent on the strength of sustained attention. We again started with a repeated measures ANOVA with a single subject excluded and then followed up with a linear mixed-effects model with all participants. As illustrated in Fig. 4B, participants were slower to initiate a saccade in low shift likelihood contexts than in high shift likelihood contexts, *F*(1,74) = 19.78, *p* < 0.001, ${\widehat{\eta }}_{p}^{2}$ = 0.211, and when *out of the zone* than when *in the zone*,* F*(1,74) =  7.99, *p* = 0.006, ${\widehat{\eta }}_{p}^{2}$ = 0.098. Most importantly, there was a significant interaction of shift likelihood by sustained attention state, *F*(1,74) =  8.74, *p* = 0.004, ${\widehat{\eta }}_{p}^{2}$ = 0.106. This interaction indicated that the cost associated with a low shift likelihood was larger when participants were *out of the zone* than when *in the zone*, suggesting that they were influenced more heavily by recent trial history when the efficacy of sustained attention was low than when it was high. A linear mixed-effects model with fixed effects of shift likelihood, sustained attention state, and their interaction, with random slopes for each main effect as well as the interaction and random intercepts, provided converging evidence (see Supplemental Table S5). We again found significant main effects of shift likelihood, *b* = -18.34, *SE* = 4.59, *t*(75.90) = -4.00, *p* < 0.001, ${R}_{p}^{2}$ = 0.004, and sustained attention state, *b* = -9.37, *SE* = 3.48, *t*(105.94) = -2.70, *p* = 0.008, ${R}_{p}^{2}$ = 0.001, as well as a significant interaction of the two factors, *b* = 9.74, *SE* = 3.61, *t*(102.77) = 2.70, *p* = 0.008, ${R}_{p}^{2}$ = 0.001. The marginal *R*^2^ was 0.005.

#### Accuracy markers of shift readiness

Given evidence of learned adjustments in flexibility and an interaction with sustained attention in the case of saccade latencies, we tested if the accuracy of target digit classifications varied according to attention state and learned shift readiness with a repeated measures ANOVA with factors of trial type (shift vs. hold), shift likelihood (low vs. high), and sustained attention state (*in the zone* vs. *out of the zone*). This analysis is important to rule out the possibility of a speed-accuracy tradeoff. For a trial to be marked as accurate, participants needed to meet gaze position criteria and make an accurate manual response (see Methods). The total number of trials per condition per participant ranged from 4 to 44 with no participants excluded. Overall, there was no evidence of a speed-accuracy tradeoff (see Fig. 4C). Participants were less accurate on shift gaze trials than on hold gaze trials, *F*(1,75) =  47.17, *p* < 0.001, ${\widehat{\eta }}_{p}^{2}$ = 0.386, and when *out of the zone* relative to periods *in the zone,* *F*(1,75) = 43.68, *p* < 0.001, ${\widehat{\eta }}_{p}^{2}$ = 0.368. No other main effects or interactions reached statistical significance. *F*s < 1.51, *p*s > 0.223.

Since our gaze criteria for hold gaze trials only required participants to make at least one fixation at the to-be-attended location between cue onset and their behavioral response, it is possible that participants also made unnecessary fixations at the to-be-ignored location on some subset of accurate trials before redirecting their gaze to the correct location. These unnecessary, extra fixations could partly account for an increase in digit categorization RT for hold trials falling in high shift likelihood blocks if they were influenced by the expectation to shift attention. We therefore tested whether the degree to which participants fixated on the to-be-ignored location prior to making a response varied based on shift likelihood and sustained attention state with another repeated measures ANOVA with factors of sustained attention state (*in the zone* vs. *out of the zone*) and shift likelihood (low vs. high). For this analysis, we included all hold attention trials with a manual response regardless of whether the participant also made an accurate fixation and/or the correct manual response, except for those trials in which the digits were replaced by an X due to an incorrect fixation appearing earlier in the trial or were excluded due to a stimulus presentation error (see Methods). The total number of trials per condition per participant ranged from 5 to 42. While the overall percentage of fixations at the to-be-ignored location did not differ according to states of sustained attention, *F*(1,75) =  0.07, *p* =  0.794, ${\widehat{\eta }}_{p}^{2}$ = < 0.001, a significant main effect of shift likelihood revealed that participants were more likely to fixate at the incorrect location prior to responding if they were in a high shift likelihood context than in a low shift likelihood context, *F*(1,75) =  127.59, *p* < 0.001, ${\widehat{\eta }}_{p}^{2}$ = 0.630 (see Fig. 4D). The interaction approached significance, *F*(1,75) = 3.73, *p* = 0.057, ${\widehat{\eta }}_{p}^{2}$ = 0.047, indicating that the shift likelihood difference in number of fixations at the to-be-ignored location trended toward being larger for *in the zone* contexts than for *out of the zone* contexts.

### Shift likelihood influences on sustained attention

Finally, we tested whether shift likelihood was associated with fluctuations in sustained attention. The VTC measure used so far accounts for the degree to which each CPT RT deviates from a participant’s mean but is insensitive to trial-by-trial dynamics. To determine whether shift likelihood influences the rate at which RTs varied over time, we calculated the standard deviation of raw RTs for correct commission CPT trials and compared across the shift likelihood contexts using a paired samples t-test to determine whether the magnitude of fluctuations in sustained attention differed based on shift likelihood. For this analysis, we marked the start of each block as the first attentional orienting cue, excluding continuous performance trials that came before the first cue of the first block in a run. We observed no significant difference in CPT RT variability across low shift likelihood (*M* = 0.13, *SD* = 0.03) and high shift likelihood (*M* = 0.13, *SD* = 0.03) contexts, *t*(75) =  1.04, *p* = 0.304, *d*_*z*_ = 0.119, indicating that participants were just as likely to fluctuate between high and low efficacy periods of sustained attention when in states of low and high shift readiness.

**Fig. 5:**
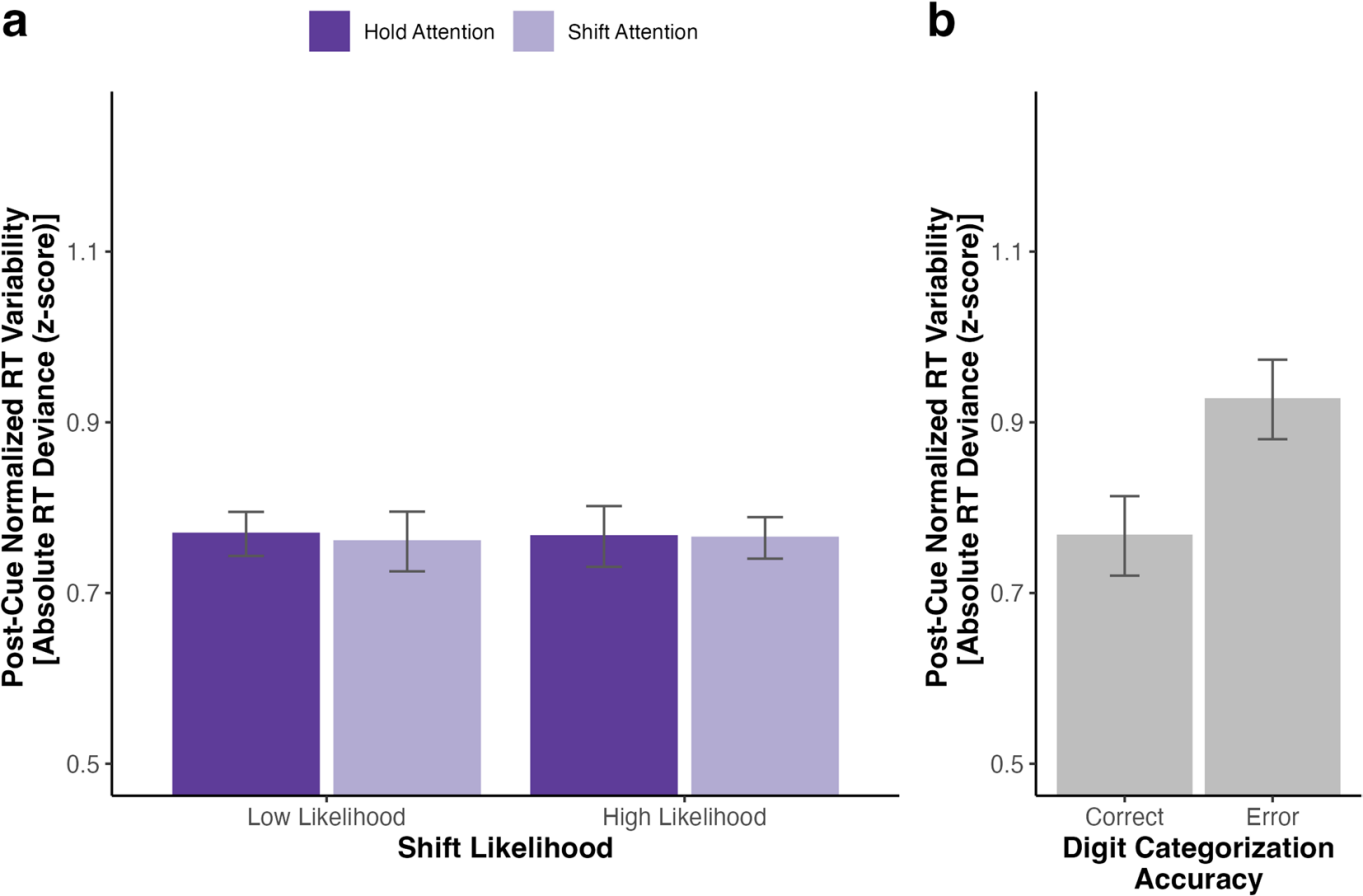
Post-attentional orienting cue non-interpolated and unsmoothed VTC as a function of (**a**) trial type and shift likelihood and (**b**) previous digit categorization accuracy. Error bars denote difference and correlation-corrected within-subject 95% confidence intervals^27^. Correlation adjustment was carried out using the Cousineau-Morey approach^28,29^.

Relatedly, as stated above, our pre-cue VTC measure of sustained attention reflected CPT RT variability both before and after each cue presentation due to interpolation and smoothing, which does not allow for causal inferences. To determine the extent to which attentional orienting trials influenced ongoing changes in CPT RT variability, we averaged the non-interpolated and unsmoothed VTC values immediately after each digit categorization period and tested for effects of previous digit categorization outcomes (see Fig. 5). For each attentional orienting trial, we averaged up to three post-cue VTC values to estimate post-cue sustained attention, excluding CPT trials that were commission errors or response omissions since these trials would not have an associated non-interpolated RT. If there were no available VTC values to average, we excluded this orienting trial from the analysis (0.59% of all trials with a post-cue period). We subjected the post-cue VTC values following accurate trials to a repeated measures ANOVA with factors of trial type (shift vs. hold) and shift likelihood (low vs. high). Post-cue variability did not differ based on trial type, *F*(1,75) = 0.23, *p* = 0.631, ${\widehat{\eta }}_{p}^{2}$ = 0.003, shift likelihood, *F*(1,75) < 0.01, *p* = 0.952, ${\widehat{\eta }}_{p}^{2}$ < 0.001, nor their interaction, *F*(1,75) = 0.10, *p* = 0.755, ${\widehat{\eta }}_{p}^{2}$ = 0.001 (see Fig. 5A). While there was no evidence that violations of shift expectations were associated with a post-cue change in sustained attention, an additional source of variability in CPT RTs may be post-error slowing following inaccurate digit categorization trials. We tested whether post-cue VTC differed based on whether participants made a correct response for the previous digit categorization using a paired samples t-test. Relative to the overall mean of each run, participants had more variable post-cue CPT RTs following a digit categorization error than after a correct response, *t*(75) = 6.84, *p*  < 0.001, *d*_*z*_ = 0.784, reflecting a decrease in the efficacy of sustained attention (see Fig. 5B).

## Discussion

In the current study, we tested the interaction of fluctuating states of sustained attention and shift readiness. As in previous studies^5,26^, we measured moment-by-moment changes in the efficacy of sustained attention that carried behavioral consequences for target detection in a CPT. Ongoing changes in arousal, as indexed by pre-cue pupil size, covaried with the efficacy of sustained attention, operationalized as CPT RT variability, and shift readiness according to a U-shaped curve with superior performance at intermediate levels of arousal. Furthermore, we manipulated the likelihood of gaze shifting over time, and individuals demonstrated robust statistical learning effects, such that the behavioral costs associated with gaze shifting in digit categorization RT and saccade latency were larger when shifting was unlikely than when shifting was highly likely^16,18^. Most interestingly, the difference in saccade latencies between low and high shift likelihood blocks was largest when participants’ sustained attention was poor, suggesting that changing states of sustained attention carry consequences for individuals’ use of probabilistic representations of shift likelihood. Additionally, we observed a difference in the number of fixations participants made at the to-be-ignored location on hold trials in the time window between cue onset and their response. Specifically, there was a robust effect such that participants generally made more fixations at the to-be-ignored location when expecting to shift gaze than when expecting to hold.

We did not find any evidence that periods of low efficacy sustained attention (high CPT RT variability) limited the degree to which individuals could harness previous learning to guide shift readiness. For example, if low efficacy sustained attention were associated with a reduction in learned shift readiness (due to decreased encoding, for instance), we would expect smaller influences of shift likelihood on digit categorization RTs and saccade latencies when CPT RT variability was high (*out of the zone*) than low (*in the zone*). Instead, the behavioral cost in saccade latency that was attributable to cue expectation was larger when sustained attention was poor. One potential explanation of this effect is that sustained attention has a protective quality that shields individuals from the behavioral disruption associated with an unexpected outcome. Control learning, such as learning to associate a context with a high or low shift likelihood, requires individuals to detect violations of expectations, use these violations to update representations of trial likelihoods, and then adjust behavior accordingly. Across diverse areas of research on cognitive control, violations of expectations, such as receiving a shift cue in a block of mostly hold cues, are associated with slower and less accurate performance^4,21,22,30–32^. Furthermore, surprising stimuli, such as a color or shape singleton, are afforded elevated attentional priority^33,34^ and spatial attention is drawn to information sources with predictable statistical structures^35^. In the context of the current study, unexpected cue stimuli may have been afforded an elevated priority that made shifting attention away from them challenging. It is possible that high efficacy sustained attention enables individuals to more quickly disengage from a salient unexpected cue, such that shift likelihood influences performance during *in the zone* periods to a lesser extent than during *out of the zone* periods.

Participants made more fixations at the to-be-ignored location when in high shift likelihood contexts than in low shift likelihood contexts. Furthermore, there was a trend that suggested this difference may be amplified when *in the zone*. However, we did not explicitly count trials with errant fixations as errors in the feedback that participants received. Although they received instructions to look wherever the color cue indicated, there was no penalty for exploring the opposing stream during the response window. Future research is needed to better understand the degree to which sustained attention may modulate learned anticipatory saccades.

The current results also highlight the degree to which fluctuations in shift readiness and sustained attention are both influenced by ongoing changes in arousal yet remain independent processes. In replication of previous studies^15^, we observed a quadratic relationship between pupil size and CPT RT variability. Likewise, we observed a quadratic relationship in the attentional orienting task that was most pronounced for hold gaze trials. Pupil size covaries with locus-coeruleus norepinephrine modulation of fronto-parietal attention regions^13,14,25^. At low levels of arousal, individuals may miss stimuli, while at high levels of arousal, individuals may be distracted by goal-irrelevant stimuli^36^. This effect may be particularly striking for hold gaze trials, which do not have the added process of overtly shifting attention. Given the larger U-shaped relationship between arousal and RT on hold trials than on shift trials, the shift cost was largest at intermediate levels of arousal. This result may partially explain why DMN activity has been linked to both high efficacy sustained attention and low attentional flexibility^5,7^. Periods of behavioral stability for sustained attention and shifting readiness may be associated with a decrease in DMN deactivation (higher activity) when arousal is at an optimal middle level^11^. While this interaction was statistically robust, the variance explained was small. Additionally, we did not find a significant linear or quadratic relationship between pre-cue pupil size and saccade latencies. While we are cautious to interpret a null result, this finding is consistent with the conclusion that arousal’s relationship with performance was stronger on trials where participants needed to hold their gaze, perhaps reflecting an added increase in stability that facilitates performance in the absence of gaze shifting. Furthermore, it is possible that the linear trend observed when predicting digit categorization RTs from pre-cue pupil size may reflect processes involved with manual response selection that would not apply to saccade latencies.

We did not observe differences in digit categorization RT shift costs across *in the zone *and *out of the zone* periods when also accounting for statistical structure, and there was no difference in post-cue CPT RT variability following shift and hold gaze trials. Together, these findings suggest that while arousal accounts for variability in both sustained attention and shift readiness, the variance shared between arousal and sustained attention and arousal and shift readiness is distinct. Likewise, participants did not differ in fluctuations of sustained attention when in high and low shift likelihood blocks. This finding is consistent with evidence that learned adjustments in shift readiness, as measured by a task-switching paradigm, are not associated with increased distractibility by flanking stimuli, suggesting that there may not be a tradeoff between shift readiness and the ability to focus on a single stimulus^37^. Therefore, sustained attention and shift readiness may be distinct facets of attentional control. This division is different than the distinction between internal and external attention^23^ since both sustained attention and shifting readiness relate to the selection of external stimuli as well as internal representations^23^. Future research will be needed to understand the neural mechanisms and behavioral consequences of arousal’s influence on both sustained attention and shift readiness.

A potential limitation of the current study is that the combination of continuous performance trials and attention orienting trials required participants to switch between two tasks. Our digit categorization RTs and saccade latencies may therefore partially reflect a switch cost as individuals stopped one task and began the next. While this would impact the raw digit categorization RTs and saccade latencies, any additional cost associated with switching tasks was constant across all conditions. Likewise, inserting colored border cues likely disrupted spontaneous changes in sustained attention to some extent. However, there were an equal number of switches between tasks per block so this aspect of the task could not bias our results. Relatedly, the real-time feedback participants received for fixation errors may have also served as an alerting cue that reduced fluctuations in sustained attention. We made this decision to help participants quickly find the to-be-attended stream and to incentivize fixating at the correct location since the colored border cues appeared at both streams. Finally, we also did not observe an interaction between shift likelihood and sustained attention in digit categorization RT shift costs. A potential explanation is that while RT shift costs are an indirect measure of shifting readiness, they are diluted by post-attentional selection processing, such as deciding the parity of the target digit.

While we did not control the luminance of the scene stimuli and gaze cues in the current study, the random orderings of scenes, use of both cue colors for all conditions, and focus on the temporal window preceding the cue onset meant that luminance could not account for our pupillometry results. Likewise, while we only included trials where the participant maintained their fixation at the to-be-attended location during the pre-cue temporal window of interest, pupil size could vary due to pupil foreshortening related to whether the participant fixated the left or right screen location. As with luminance, any differences in pupil size based on the fixated location would be randomly distributed across conditions.

The current results are important for understanding the interaction of attentional fluctuations in healthy cognition, as well as in disorders of control. In particular, our results suggest that while sustained attention and shift readiness result from dissociable control operations, studying one domain alone presents an incomplete characterization of behavior. Moment-by-moment variation in shift readiness varies in part according to the statistical structure of the environment, but this relationship is amplified under conditions of poor sustained attention. An important topic for future research will be whether disorders associated with impairments of sustained attention, such as attention deficit hyperactivity disorder, may be associated with an increased reliance on trial history in setting moment-by-moment shift readiness. Likewise, sustained attention abilities and the strategies used in CPTs vary across the lifespan^38^. An area for future research is therefore whether children and older adults, both of whom have reduced sustained attention abilities compared to young and middle-aged adults, are more heavily influenced by shift likelihood statistical structure.

In the current study, we tested the interaction of fluctuations in sustained attention and shift readiness. While both the strength of sustained attention and shifting readiness varied with arousal, as measured by pupil size, fluctuations in sustained attention were not associated with overall changes in shift readiness. Instead, signatures of shift readiness were smaller for saccade latencies when participants were *in the zone* than when *out of the zone*, potentially reflecting a benefit for strong sustained attention in overcoming unexpected stimuli.

## Methods

### Participants

Ninety-nine individuals (54 women, 42 men, 3 preferred not to respond), ranging in age from 18 to 40 years (*M* = 19.3, *SD* = 2.59) completed the study in exchange for a $10 Amazon gift card or for course credit. We conducted a simulation-based power analysis using the R package, Superpower, to determine the number of participants we would need to reach 95% power for finding the interaction of cue type (shift gaze vs. hold gaze) and shift likelihood (low vs. high). We based this analysis off of the means and correlations among conditions of a previous study of learned attentional flexibility (see Experiment 3)^16^. This simulation indicated that we would need five participants to reach 95% power. However, the goals of the current study were to examine the interaction of this learning effect with CPT performance. Previous studies using the single stream gradual CPT have included 20–30 participants^5,11^. Given our design, we could not control the number of rare trials (e.g., a shift cue appearing in a low shift likelihood block) that would appear while participants had high or low CPT RT variability and we thus anticipated having a small number of trials for some conditions. To address this added noise, we increased the sample size approximately threefold.

The study was approved by the Wake Forest University Institutional Review Board and was performed in accordance with the Declaration of Helsinki. All participants signed an approved informed consent form. Participants reported normal or corrected-to-normal vision, normal color vision, no history of psychiatric or neurological disease, no history of concussion within the past 12 months, and no current use of psychoactive medications. Twenty-three participants were excluded from our analyses for having overall behavioral accuracies (computed according to manual response and eye gaze criteria) below 70% (n =  12), not completing at least three runs of the experimental task (n = 2), an inability to track eye position (n = 7), or experiencing technical difficulties during participation (n = 2). For a single participant, we excluded one run during which they made no CPT responses.

### Apparatus

Participants completed the study in a dimly lit room. Stimulus presentation was controlled by the Psychophysics Toolbox^39^ (version 3.0.18) running in MATLAB. All stimuli were displayed on a Dell U2415 monitor that was positioned 91.4 cm from the participant. The participant’s head was stabilized with a chin rest and all responses were made with a Logitech F310 game controller. We tracked eye gaze position with an Eyelink 1000 recording at 1000 Hz and participants completed a calibration procedure prior to the start of each run of the task. We tracked the gaze position and pupil size of the right eye for all participants.

### Measures

Prior to completing the task, participants provided demographic information and completed a series of self-report measures (not reported here), consisting of: the Barratt Impulsiveness Scale Version 11 (BIS-11)^40^, the DSM-5 self-rated Level 1 cross-cutting symptom measure—Adult^41^, the Generalized Anxiety Disorder Version 7 (GAD-7)^42^, the Patient Health Questionnaire Version 9 (PHQ-9)^43^, the State-Trait Anxiety Inventory (STAI)^44, and^ the World Health Organization Disability Assessment Schedule 2.0 (WHODAS)^45^.

### Stimuli and procedure

Participants completed a variant of the gradual CPT, with embedded spatial orienting cues that signaled them to either maintain fixation at the current location or to make a saccade. The display consisted of two continuous streams of black and white city and mountain scenes, which appeared inside two circular apertures (diameter = 9.7° visual angle) positioned on the left and right sides of the screen (center-to-center eccentricity 13.5° visual angle). The stimuli consisted of 10 city and 10 mountain scenes, as well as 10 phase scrambled scenes, all of which have been used in previous studies^5,11,26^. Stimulus presentations were synced to the monitor’s refresh rate of 59.95 Hz. To ease interpretation, we report all stimulus timing as if the monitor’s refresh rate here and throughout the paper was 60 Hz, thereby rounding to the nearest multiple of 100 ms in the ranges reported. The images in both streams gradually changed approximately every 800 ms. We divided the 800 ms interval into 16 steps during which the coherence of one image decreased while the next image’s coherence increased, using a linear pixel-by-pixel interpolation, producing a gradual change in images at both the left and right stream locations.

Participants were instructed to fixate on one of the two stream locations and press a designated button with their right thumb each time that they were confident that the image at the to-be-attended location was a city scene. They were instructed to withhold presses when the image was a mountain scene. Participants never needed to attend to both streams simultaneously and were told to fixate on the cued stream alone. The majority of images presented at the to-be-attended location over the course of the task were city scenes (*M* = 89.28%, *SD* = 0.16%). Participants continued responding to city scenes and withholding responses to mountains at the attended location for a variable interval that approximately ranged between 4000 and 8800 ms, with the last image in the sequence at both streams consisting of a scrambled scene, which participants were to ignore.

The attentional orienting task was embedded directly in the gradual onset streams such that there was a continuous onset of new images. After a series of CPT trials, participants received a cue to either hold fixation at the current location or to make a saccadic eye movement at the start of the image transition following the scrambled image described above. The orienting cue was a colored border that appeared around each of the stream locations, regardless of the currently fixated location. Participants were to attend to the left stream if the border was blue (RGB = [142 171 213], luminance = 95.9 cd/m^2^) and the right stream if the border was pink (RGB = [199 142 213], luminance = 87.3 cd/m^2^). Thus, if a participant received a blue border while fixating on the right stream, they would be cued to make a saccade to the left location. However, if they received a blue border while they were already fixating on the left stream, it would signal them to hold their gaze at the current location. At the same time as the colored border onset, randomly selected digits, ranging from 1 to 8, with the restriction that the digits could not be identical, appeared centered at each stimulus location. The digits and colored borders remained on the screen for approximately 2400 ms and scrambled scene images continued to gradually change behind the target stimulus at the same rate as the CPT. Participants made a manual response, pressing a button with their left or right index fingers, to indicate whether the target digit at the cued location was odd or even, respectively. At the conclusion of the attention cue window, the colored borders and digits offset and the images inside each aperture returned to city and mountain scenes.

A stimulus presentation error affected the last trial of each run such that the trial ended prematurely during the last stage of image transition for some participants (n=29 among those retained in the final analysis) or the response was not recorded for the remaining participants (n=47 among those retained in the final analysis). We exclude any of these trials where no response was made or recorded because the failure to record a press could have resulted from the error. However, we include trials with an accurate press since any recorded press would have occurred prior to the premature trial end. Alternatively, excluding all final trials from each run produced the same pattern of results and did not change any conclusions.

As in previous tests of learned attentional flexibility^16–18^, we manipulated the frequency of gaze shift cues over time. Specifically, we divided each run into four blocks of 12 trials each. Within a run, two of these blocks were randomly selected to have a high likelihood of shifting attention, while the other two had a low likelihood of shifting. Due to a programming error that mislabeled the starting location at the beginning of some blocks, the probability of receiving a shift cue varied between 16.67 and 33.33% in low-likelihood blocks, and between 66.67 and 83.33% in high-likelihood blocks. To provide participants with real-time information about the accuracy of their gaze position, we divided the display in half along the vertical meridian and warned participants if at least one gaze sample was detected on the wrong side of the meridian since the last attention cue window. In this case, instead of seeing two digits appear during the subsequent attention window, an X appeared inside each aperture and the trial was counted as an error. These X warnings did not stop the continuous progression of stimuli. Critically, the border color changed as on normal trials so that participants were given an indication of the correct location to fixate. Given the programming error described above, participants sometimes received an erroneous X gaze warning for the first trial of a block, causing that trial to be counted as an inaccurate response in the block-ending feedback. We exclude these rare, mislabeled trials from all digit categorization RT and accuracy analyses regardless of the participant’s gaze position. This procedure resulted in a reduction of 2.05% of all digit categorization trials across the entire sample, with 1–7 digit categorization trials excluded per participant (*M* = 3.82, *SD* = 1.56).

We divided the task into runs of 48 attention cue trials each and participants completed three or four runs of the task depending on the time available within a 1-h study session. Each run lasted approximately seven minutes and participants received feedback on their accuracy for parity judgments only at the conclusion of the run. Participants completed a scaffolded practice prior to the main task.

### Data analysis

Data analysis was conducted in MATLAB (MATLAB, 2022), R (version 4.4.2), and DataViewer (SR Research Ltd., version 4.4.1) software. For the analysis of RTs and saccade latencies, only accurate trials were considered. For all analyses, we recoded response accuracy to include gaze metrics in addition to whether participants had made the correct parity response. Specifically, for hold gaze trials, we required that participants make at least one fixation at the to-be-attended location during the temporal window ranging from cue onset through the participant’s manual response, resulting in the exclusion of 0.50% of all potential hold trials from RT analyses. Similarly, for shift gaze trials, a trial was coded as accurate only if the participant made a saccade from the correct starting interest area to the other interest area in the temporal window between cue onset and manual response, resulting in the exclusion of 4.27% of all potential shift trials from RT and saccade latency analyses. In both cases, the interest areas matched the on-screen locations of the left and right streams. This definition of accuracy differed from that used to provide participants feedback, which was based on manual response accuracy alone (see above). Trials with an inaccurate response, no response, or that were marked incorrect due to a pre-cue fixation on the wrong side of the display consisted of 8.10% of all trials.

For RT and saccade latency analyses, we trimmed outlier responses that were less than 200 ms for RTs and less than 50 ms for saccade latencies, resulting in a reduction of 0% and less than 1% of trials with an accurate response for RT and saccade latency trials, respectively. We ran repeated measures ANOVAs and linear mixed-effects models using the afex^46^ package in R. All raw behavioral data, eye tracking data, and code for stimulus presentation, data analysis, and figure creation are available at: https://osf.io/av8fb/

#### Variance time course

Response times for CPT trials were computed using an iterative algorithm as in previous studies with respect to the start of each image’s gradual onset^5^. For example, an RT of 720 ms would signify a press made at 90% image coherence of the onsetting image and 10% coherence of the offsetting image. We first assigned unambiguous presses such that presses made at 80% image coherence or higher were assigned to the onsetting image and presses made at 40% image coherence or lower were assigned to the offsetting image. We assigned ambiguous presses between these points in time to trials according to an iterative algorithm (accounting for 28.57% of all non-scrambled image CPT trials). In these cases of ambiguity, we first assigned the press to whichever image did not already have an assigned press. If neither image had an assignment, we attributed the press to the adjacent image that was closest to 100% coherent (onsetting image >  = 60% coherent, offsetting image < 60% coherent) unless one of the images was a mountain scene or the beginning or end of an attention cue window. In these cases, we assigned the press to the adjacent image that was not a mountain or scrambled image. For trials with multiple presses, we assigned RTs based on time, with the fastest RTs assigned first. On average, only 1.39% (*SD* = 1.47) of all presses per participant were left unassigned (range 0–10.55%) when excluding rare presses affected by a stimulus timing imperfection (see below), indicating that, overall, there was a strong correspondence between presses made and CPT trials.

As a measure of sustained attention, we calculated the Variance Time Course (VTC) for each participant per run of the task as in previous studies^5,11,26.^ To estimate RT changes for approximately every 800 ms throughout the run, including trials where the participant did not make a response (correct omissions for mountains or misses for cities) and during the frames of the attention orienting task, we used linear interpolation based on the two neighboring RTs. Next, we z-transformed the time course so that it represented how far each RT deviated from the mean of the actual data, not accounting for the interpolated values, and took the absolute value. Given the high level of noise in RT measurements, we filtered the time course with a Gaussian window of 20 observations. Finally, we proceeded with a median split of values in the smoothed time course to define *in the zone* and *out of the zone* epochs relative to each participant’s own run-by-run performance. For 13 participants, stimulus presentation of the city and mountain streams prior to the first attention orienting cue was not reliably fixed at 50 ms per frame. For these participants, we started the VTC after the first digit categorization trial and have excluded the first digit categorization trial from the analysis. For all other trials, across all participants, we detected a discrepancy between CPT RT and the degree of image coherence due to stimulus timing imperfections on 0.01% of CPT trials. We excluded these CPT trials from all RT and accuracy analyses. We also excluded any CPT trials with an ambiguous press that came immediately before or after the affected trial, extending forward in time until an unambiguous press was detected or a digit categorization trial began. Following a timing imperfection, we retained trials where an ambiguous press was assigned to the offsetting image as long as an unambiguous press was assigned to the corresponding onsetting image. Exclusion of a single trial fitting this retention rule did not change any conclusions. In total, this procedure resulted in a reduction of fewer than one trial per participant on average (*M* = 0.18, *SD* = 0.58), and RTs for these trials were then interpolated as above. We retained any additional rare trials where a stimulus timing imperfection occurred but there was either no RT or an RT that was consistent with the expected coherence level.

#### Pupillometry

We recorded pupil size throughout the experimental task. For each digit categorization trial, we defined a temporal window beginning 500 ms prior to the onset of the shift or hold cue and continuing until cue onset Although our task required participants to shift gaze position, thus potentially leading to pupil foreshortening effects that were dependent on the current to-be-attended location, any such effects were randomly distributed across all conditions and therefore cannot account for the current results. Preprocessing of the pupillometry data was conducted in the R package pupilPre (0.6.2). We first removed samples with gaze positions falling outside the boundaries of the display and those flagged by the Eyelink as falling inside of a blink. For a window of 100 ms before the beginning of the marked blink and continuing 100 ms after the end of the marked blink, we calculated the difference in pupil size across sequential samples. If this difference was greater than five units, the sample was removed. Lastly, if there were fewer than six remaining samples that were flanked by missing or removed data by at least two samples on either side, we also excluded these intermediate samples. For digit categorization trials meeting the criteria described above with at least 50 samples left after preprocessing inside the 500 ms window, we averaged all remaining samples and z-transformed the averages across runs to provide a trial-by-trial measure of pupil size. This procedure resulted in a reduction of only 10 trials across the entire sample after applying the other digit categorization trial retention criteria. Furthermore, for all pupil size analyses, we excluded any trials (0.21% of all trials) where there were otherwise valid pupil size data (see Methods) but the participant did not maintain fixation at the accurate location during the 500 ms pre-cue window defined above. We conducted the z-transformation according to the mean and standard deviation of pupil sizes across all retained hold gaze and shift gaze trials for all analyses, meaning that the data were centered with respect to the full dataset instead of just the included trials for the saccade latency analysis.

#### Linear mixed-effects models

In all linear mixed-effects models, we first fit a model with the maximal random effects structure^47^. However, in all cases, the maximal model led to either a convergence failure or a singular fit. Therefore, we iteratively simplified the random effects structure by removing the estimated correlation between random effects, followed by each random slope, starting with the highest order effects, and followed by those with the least variance, until we found a model without convergence or singularity warnings. We detail below this procedure for each model tested. In all cases, we used the restricted maximum likelihood (REML) method, used sum contrasts for categorical factors, mean-centered continuous predictors, and calculated p-values according to Satterthwaite’s method. As a measure of effect size, we report the proportion of variance in the outcome variable that was uniquely explained by each fixed effect, the semi-partial *R*^2^ (${R}_{p}^{2}$), as well as the marginal *R*^2^, the total proportion of variance explained by the fixed effects, using the *R* package *r2glmm* and the Nakagawa and Schielzeth approach^48^.

In our first model, we predicted ongoing changes in the efficacy of sustained attention, as indexed by the averaged pre-cue smoothed variance time course, with linear and quadratic fixed effects of pupil size. After the full maximal model failed to converge, we simplified the random effects structure by removing the correlations among random intercepts and slopes: $VTC \sim 1+ PupilSize+{PupilSize}^{2}+(1+PupilSize+{PupilSize}^{2}|\left| participant\right)$.

Next, we predicted digit parity classification RTs with fixed effects of cue type, linear and quadratic trends in pupil size, and the interactions between the cue type and pupil size effects. When the maximal model did not successfully converge, we again removed the correlations among random intercepts and slopes and then simplified again by removing the random slope associated with the interaction of cue type by the quadratic pupil size effect: $$RT \sim 1+ CueType+ PupilSize+{PupilSize}^{2}+ CueType* {PupilSize+CueType*PupilSize}^{2}$$$$+(1+CueType+ PupilSize+{PupilSize}^{2}+ CueType* {PupilSize} |\left| participant\right)$$.

We used a similar model to account for saccade latencies according to linear and quadratic fixed effects of pupil size. After the maximal model yielded a singular fit, we simplified the random effects structure by removing the estimated correlations among random intercepts and slopes: $SaccadeLatency \sim 1+ PupilSize+{PupilSize}^{2}+(1 +PupilSize + PupilSize^2 |\left| participant\right)$.

We predicted digit parity categorization RTs on hold and shift trials according to fixed effects for the main effects and interactions of cue type (shift vs. hold), shift likelihood (low vs. high), and sustained attention state (*in the zone* vs. *out of the zone*). The maximal model again yielded a singular fit. We simplified the random effects structure by first removing correlations among random intercepts and slopes, followed by removing random slopes for the three-way interaction, random slopes for the interaction of cue type by shift likelihood, and random slopes for the interaction of cue type by sustained attention state after each of these models also produced singular fits. The final model contained random intercepts and random slopes for each main effect: $$\begin{aligned} & RT \sim 1+CueType*ShiftLikelihood* State(1+CueType+ShiftLikelihood+State || participant).\end{aligned}$$

Finally, we predicted saccade latencies according to fixed effects of shift likelihood, sustained attention state, and their interaction. When the maximal model produced a singular fit, we removed the correlations among random intercepts and slopes such that the model included random intercepts and random slopes for shift likelihood, sustained attention state, and their interaction: $SacLatency \sim 1+ShiftLikelihood+State+ShiftLikelihood*State+(1+ShiftLikelihood+State+ShiftLikelihood*State || participant).$

## Supplementary Information

Below is the link to the electronic supplementary material.

Supplementary Material 1

## Data Availability

All raw behavioral data, eye tracking data, and code for stimulus presentation, data analysis, and figure creation are available at: https://osf.io/av8fb/
