# Correction: Learned saccade readiness varies with fluctuations in sustained attention

**DOI:** 10.1038/s41598-026-57612-0

**Published:** 2026-07-24

**Authors:** Anthony W. Sali, Madison P. Shaver, Anna B.Toledo, Austin L.Torain, Isabel N. Flicker

**Affiliations:** Department of Psychology, Wake Forest University, 1834 Wake Forest Road, Winston-Salem, NC 27109 USA

Correction to: *Scientific Reports* 10.1038/s41598-025-14340-1, published online 23 August 2025

The original version of this Article was revised. Due to a stimulus presentation error, the last trial of each run ended prematurely during the final stage of image transition for some participants (n = 29 among those retained in the original paper’s analysis) or the response was not recorded for the remaining participants (n = 46 among those retained in the original paper’s analysis).

The trials where no response was made or recorded have now been excluded because they could have resulted from the error. However, trials with an accurate press are still included, since any recorded press would have occurred prior to the premature trial end. Excluding all final trials from each run produced the same pattern of results and did not change any conclusions.

Additionally, the calculation of excluded trial percentages and the number of phase scrambled scenes used in the task, both of which did not affect any analyses, were corrected.

Furthermore, the calculation of errant fixations were corrected due to the original calculation having missed some fixations.

Finally, the percentage of errant fixations on hold trials is now calculated using only trials on which participants made a manual response, reflecting a more appropriate operationalization of the measure. In the corrected analysis, the previously significant interaction of shift likelihood by sustained attention state remained in the same direction as originally reported but was trending at *p* = 0.057. Consequently, the Results and Discussion to qualify this finding have been revised.

After recalculating overall accuracies, one participant who was previously excluded for accuracy below 70% now exceeded this threshold. As a result, this participant was included in all analyses. Given this change, in two linear mixed effects models a random slope was added or removed according to the rules outlined in our original analysis. With the exception of the errant fixation interaction described above, all conclusions from the original paper remain unchanged.

As a result of these change the main text was adjusted as follows:

Under the Results section, subsection “Linking arousal, sustained attention, and shift readiness” the text was corrected from.

“In support of previous studies^15^, we observed a significant quadratic relationship with the lowest VTC values at intermediate pupil sizes and higher VTC values (poorer sustained attention) as pupil size deviated, *b* = 0.012, *SE* = 0.002, *t*(106.71) = 6.55, *p* < 0.001, *R*^2^_*p*_ = 0.006. There was no significant linear effect, *b* = 0.004, *SE* = 0.003, *t*(79.40) = 1.19, *p* = 0.237, *R*^2^_*p*_ < 0.001 and the marginal *R*^*2*^ was 0.007.”

to now read:

“In support of previous studies^15^, we observed a significant quadratic relationship with the lowest VTC values at intermediate pupil sizes and higher VTC values (poorer sustained attention) as pupil size deviated, *b* = 0.012, *SE* = 0.002, *t*(108.59) = 6.57, *p* < 0.001, *R*^2^_*p*_ = 0.006. There was no significant linear effect, *b* = 0.003, *SE* = 0.003, *t*(80.30) = 1.06, *p* = 0.294, *R*^2^_*p*_ < 0.001 and the marginal *R*^*2*^ was 0.007.”

In the same subsection under Figure 2:

“As illustrated in Fig. 2B, we tested the relationship between pre-cue pupil size and digit categorization RTs with a linear mixed-effects model containing fixed effects of cue type (shift vs. hold), linear and quadratic pupil size, and the interactions between cue type and pupil size effects (marginal *R*^*2*^ = 0.124). The random effects structure included random slopes for cue type, the linear and quadratic effects of pupil size, the interactions between the cue type and pupil size effects, and random intercepts. Participants were slower on shift trials than on hold trials, *b* = − 136.84, *SE* = 6.32, *t*(88.93) = − 21.66, *p* < 0.001, *R*^2^_*p*_ = 0.096, and there were linear, *b* = 9.71, *SE* = 4.24, *t*(77.17) = 2.29, *p* = 0.025, *R*^2^_*p*_ = 0.001, and quadratic,* b* = 9.41, *SE* = 2.33, *t*(66.01) = 4.04, *p* < 0.001, *R*^2^_*p*_ = 0.002, effects of pupil size on digit categorization RT (see Supplemental Table S2). However, these effects were qualified by a significant interaction of cue type and the quadratic term, *b* = 4.85, *SE* = 1.96, *t*(101.92) = 2.47, *p* = 0.015, *R*^2^_*p*_ < 0.001, as digit categorization RTs for hold gaze trials varied according to a U-shape more strongly than did shift trials. The linear effect did not vary according to cue type, *b* = 1.00, *SE* = 3.55, *t*(76.77) = 0.28, *p* = 0.779, *R*^2^_*p*_ < 0.001. To test the relationship between pretrial pupil size and saccade latencies for each type of attentional orienting trial, we used another linear mixed-effects model of saccade latencies with fixed effects of linear and quadratic pupil size, random slopes for the linear effect, and random intercepts (marginal *R*^*2*^ < 0.001; see Fig. 2C). There were no significant linear, *b* = 4.84, *SE* = 3.39, *t*(78.58) = 1.43, *p* = 0.157, *R*^2^_*p*_ < 0.001, or quadratic, *b* = − 0.48, *SE* = 1.80, *t*(3276.17) = − 0.27, *p* = 0.787, *R*^2^_*p*_ < 0.001, relationships between pretrial pupil size and saccade latency (see Supplemental Table S3).”

Now reads:

“As illustrated in Fig. 2B, we tested the relationship between pre-cue pupil size and digit categorization RTs with a linear mixed-effects model containing fixed effects of cue type (shift vs. hold), linear and quadratic pupil size, and the interactions between cue type and pupil size effects (marginal *R*^*2*^ = 0.122).The random effects structure included random slopes for cue type, the linear and quadratic effects of pupil size, the interaction between cue type and the linear pupil size effect, and random intercepts. Participants were slower on shift trials than on hold trials *b* = -136.60, *SE* = 6.26,* t*(90.91) = -21.82, *p* < 0.001, *R*^2^_*p*_ = 0.095, and there were linear, *b* = 9.94, *SE* = 4.19, *t*(78.51) = 2.37,* p* = 0.020, *R*^2^_*p*_ = 0.001, and quadratic, *b* = 9.10, *SE* = 2.30, *t*(66.51) = 3.95, *p* < 0.001, *R*^2^_*p*_ = 0.002, effects of pupil size on digit categorization RT (see Supplemental Table S2). However, these effects were qualified by a significant interaction of cue type and the quadratic term, *b* = 5.01, *SE* = 1.92, *t*(2,422.86) = 2.61, *p* = 0.009, *R*^2^_*p*_ = 0.001, as digit categorization RTs for hold gaze trials varied according to a U-shape more strongly than did shift trials. The linear effect did not vary according to cue type, *b* = 1.38, *SE* = 3.54, *t*(79.21) = 0.39, *p* = 0.698, *R*^2^_*p*_ < 0.001. To test the relationship between pretrial pupil size and saccade latencies for each type of attentional orienting trial, we used another linear mixed-effects model of saccade latencies with fixed effects of linear and quadratic pupil size, random slopes for the linear and quadratic effects, and random intercepts (marginal *R*^*2*^ < 0.001; see Fig. 2C). There were no significant linear, *b* = 4.76, *SE* = 3.35, *t*(79.43) = 1.42, *p* = 0.158, *R*^2^_*p*_ < 0.001, or quadratic, *b* = -0.43, *SE* = 1.80, *t*(26.44) = -0.24, *p* = 0.812, *R*^2^_*p*_ < 0.001, relationships between pretrial pupil size and saccade latency (see Supplemental Table S3).”

Under the subsection “The interaction of sustained attention and learned shift readiness” the sentence:

“As expected, individuals made significantly more commission, *t*(74) = 12.31, *p* < 0.001, *d*_*z*_ = 1.421, and more omission, *t*(74) = 6.27, *p* < 0.001 *d*_*z*_ = 0.724, errors when *out of the zone* than when *in the zone*, suggesting that our VTC measure accounted for fluctuations in sustained attention (see Fig. 3B,C).”

Now reads:

“As expected, individuals made significantly more commission, *t*(75) = 12.49, *p* < 0.001, *d*_*z*_ = 1.433, and more omission, *t*(75) = 6.37, *p* < 0.001 *d*_*z*_ = 0.731, errors when *out of the zone* than when *in the zone*, suggesting that our VTC measure accounted for fluctuations in sustained attention (see Fig. 3B,C).”

In the same subsection under Figure 3:

“As illustrated in Fig. 4A, participants were slower on shift gaze trials than on hold gaze trials, *F*(1,73) = 397.76, *p* < 0.001, ${\widehat{\eta }}_{p}^{2}$= 0.845, and in blocks with a high shift likelihood than those with a low shift likelihood, *F*(1,73) = 51.33, *p* < 0.001, ${\widehat{\eta }}_{p}^{2}$ = 0.413. The cost in digit categorization RT associated with shifting was significantly smaller in high shift likelihood blocks than in low shift likelihood blocks, *F*(1,73) = 141.36, *p* < 0.001, ${\widehat{\eta }}_{p}^{2}$= 0.659, indicating learned adjustments in shift readiness. In addition to this evidence of learned shift readiness, we also observed a general cost associated with *out of the zone* periods such that participants were slower to respond than when *in the zone*, *F*(1,73) = 6.06, *p* = 0.016, ${\widehat{\eta }}_{p}^{2}$= 0.077, regardless of cue type and shift likelihood. However, learned shift readiness did not differ for *in the zone* and *out of the zone* epochs, as indicated by the lack of a three-way interaction, *F*(1,73) = 1.10, *p* = 0.297, ${\widehat{\eta }}_{p}^{2}$ = 0.015. No other interactions reached significance, *F*s < 3.43, *p*s > 0.068.

To test the robustness of our findings from the ANOVA, we compared these results to those from a linear mixed-effects model with fixed effect regressors for the main effects and interactions of trial type (shift vs. hold), shift likelihood (low vs. high), and state (*in the zone* vs. *out of the zone*), as well as random slopes for each main effect and random intercepts (see Supplemental Table S4). As in the ANOVA, there were significant main effects of trial type, b = − 114.34, SE = 6.03, *t*(82.35) = − 18.98,* p* < 0.001, *R*^2^_*p*_ = 0.071, shift likelihood, *b* = 35.43, *SE* = 4.22, *t*(81.22) = 8.39, *p* < 0.001, *R*^2^_*p*_ = 0.007, and sustained attention state, *b* = -9.92, *SE* = 4.06, *t*(109.55) = -2.44, *p* = 0.016, *R*^2^_*p*_ = 0.001. A significant interaction of trial type by shift likelihood, *b* = 40.88, *SE* = 3.33, *t*(11,848.14) = 12.27, *p* < 0.001, *R*^2^_*p*_ = 0.010, again provided evidence of learned shift readiness. However, learned shift readiness did not differ for *in the zone* and *out of the zone* epochs, as indicated by the lack of a three-way interaction, *b* = − 2.26, *SE* = 3.36, *t*(11,874.70) = − 0.67, *p* = 0.500, *R*^2^_*p*_ < 0.001, reflecting no change in shift readiness with fluctuations in sustained attention. The remaining interactions also failed to reach significance, *t*s < 1.92, *p*s > 0.056 and the marginal R^2^ was 0.137.”

Now reads:

“As illustrated in Fig. 4A, participants were slower on shift gaze trials than on hold gaze trials, *F*(1,74) = 393.08, *p* < 0.001, ${\widehat{\eta }}_{p}^{2}$ = 0.842, and in blocks with a high shift likelihood than those with a low shift likelihood, *F*(1,74) = 53.73, *p* < 0.001, ${\widehat{\eta }}_{p}^{2}$ = 0.421. The cost in digit categorization RT associated with shifting was significantly smaller in high shift likelihood blocks than in low shift likelihood blocks, *F*(1,74) = 146.20, *p* < 0.001, ${\widehat{\eta }}_{p}^{2}$ = 0.664, indicating learned adjustments in shift readiness. In addition to this evidence of learned shift readiness, we also observed a general cost associated with *out of the zone* periods such that participants were slower to respond than when *in the zone*,* F*(1,74) = 6.20, *p* = 0.015, ${\widehat{\eta }}_{p}^{2}$= 0.077, regardless of cue type and shift likelihood. However, learned shift readiness did not differ for *in the zone* and *out of the zone* epochs, as indicated by the lack of a three-way interaction, *F*(1,74) = 1.37, *p* = 0.246, ${\widehat{\eta }}_{p}^{2}$ = 0.018. No other interactions reached significance, *F*s < 3.15, *p*s > 0.080.

To test the robustness of our findings from the ANOVA, we compared these results to those from a linear mixed-effects model with fixed effect regressors for the main effects and interactions of trial type (shift vs. hold), shift likelihood (low vs. high), and state (*in the zone* vs. *out of the zone*), as well as random slopes for each main effect and random intercepts (see Supplemental Table S4). As in the ANOVA, there were significant main effects of trial type,* b* =—113.74, *SE* = 5.98, *t*(83.71) = -19.03,* p* < 0.001, *R*^2^_*p*_ = 0.069, shift likelihood, *b* = 35.73, *SE* = 4.18, *t*(82.75) = 8.54, *p* < 0.001, *R*^2^_*p*_ = 0.007, and sustained attention state, *b* = -9.82, *SE* = 4.03, *t*(111.81) = -2.44, *p* = 0.016, *R*^2^_*p*_ = 0.001. A significant interaction of trial type by shift likelihood, *b* = 41.09, *SE* = 3.32, *t*(11,977.91) = 12.39, *p* < 0.001, *R*^2^_*p*_ = 0.010, again provided evidence of learned shift readiness. However, learned shift readiness did not differ for *in the zone* and *out of the zone* epochs, as indicated by the lack of a three-way interaction, *b* = -2.50, *SE* = 3.34, *t*(12,004.23) = -0.75, *p* = 0.454, *R*^2^_*p*_ < 0.001, reflecting no change in shift readiness with fluctuations in sustained attention. The remaining interactions also failed to reach significance, *t*s < 1.88, *p*s > 0.060 and the marginal *R*^*2*^ was 0.135.”

In the “Gaze markers of shift readiness” subsection:

“As illustrated in Fig. 4B, participants were slower to initiate a saccade in low shift likelihood contexts than in high shift likelihood contexts, *F*(1,73) = 18.62, *p* < 0.001, ${\widehat{\eta }}_{p}^{2}$ = 0.203, and when *out of the zone* than when *in the zone*, *F*(1,73) = 7.94, *p* = 0.006, ${\widehat{\eta }}_{p}^{2}$= 0.098. Most importantly, there was a significant interaction of cue type by sustained attention state, *F*(1,73) = 8.29, *p* = 0.005, ${\widehat{\eta }}_{p}^{2}$= 0.102. This interaction indicated that the cost associated with a low shift likelihood was larger when participants were *out of the zone* than when *in the zone*, suggesting that they were influenced more heavily by recent trial history when the efficacy of sustained attention was low than when it was high. A linear mixed-effects model with fixed effects of shift likelihood, sustained attention state, and their interaction, with random slopes for each main effect as well as the interaction and random intercepts, provided converging evidence (see Supplemental Table S5). We again found significant main effects of shift likelihood, *b* = − 17.97, *SE* = 4.62, *t*(74.74) = − 3.89, *p* < 0.001, *R*^2^_*p*_ = 0.004, and sustained attention state, *b* = − 9.51, *SE* = 3.50, *t*(103.87) = − 2.72, *p* = 0.008, *R*^2^_*p*_ = 0.001, as well as a significant interaction of the two factors, *b* = 9.68, *SE* = 3.64, *t*(100.69) = 2.66, *p* = 0.009, *R*^2^_*p*_ = 0.001. The marginal *R*^*2*^ was 0.005.”

Now reads:

“As illustrated in Fig. 4B, participants were slower to initiate a saccade in low shift likelihood contexts than in high shift likelihood contexts, *F*(1,74) = 19.78, *p* < 0.001, ${\widehat{\eta }}_{p}^{2}$ = 0.211, and when *out of the zone* than when *in the zone*, *F*(1,74) = 7.99, *p* = 0.006, ${\widehat{\eta }}_{p}^{2}$= 0.098. Most importantly, there was a significant interaction of shift likelihood by sustained attention state, *F*(1,74) = 8.74, *p* = 0.004, ${\widehat{\eta }}_{p}^{2}$= 0.106. This interaction indicated that the cost associated with a low shift likelihood was larger when participants were *out of the zone* than when *in the zone*, suggesting that they were influenced more heavily by recent trial history when the efficacy of sustained attention was low than when it was high. A linear mixed-effects model with fixed effects of shift likelihood, sustained attention state, and their interaction, with random slopes for each main effect as well as the interaction and random intercepts, provided converging evidence (see Supplemental Table S5). We again found significant main effects of shift likelihood, *b* = -18.34, *SE* = 4.59, *t*(75.90) = -4.00,* p* < 0.001, *R*^2^_*p*_ = 0.004, and sustained attention state, *b* = -9.37, *SE* = 3.48, *t*(105.94) = -2.70, *p* = 0.008, *R*^2^_*p*_ = 0.001, as well as a significant interaction of the two factors, *b* = 9.74, *SE* = 3.61, *t*(102.77) = 2.70, *p* = 0.008, *R*^2^_*p*_ = 0.001. The marginal *R*^*2*^ was 0.005.”

In the “Accuracy markers of shift readiness” subsection:

“Overall, the results paralleled those found with RTs with no evidence of a speed-accuracy tradeoff (see Fig. 4C). Participants were less accurate on shift gaze trials than on hold gaze trials, *F*(1,74) = 74.48, *p* < 0.001, ${\widehat{\eta }}_{p}^{2}$= 0.502, and when *out of the zone* relative to periods *in the zone,* *F*(1,74) = 37.11, *p* < 0.001, ${\widehat{\eta }}_{p}^{2}$= 0.334. We also again observed larger shift costs in blocks associated with a low shift likelihood than in those associated with a high shift likelihood, *F*(1,74) = 4.30, *p* = 0.042, ${\widehat{\eta }}_{p}^{2}$= 0.055, providing additional evidence of learned shift readiness. No other main effects or interactions reached significance, *F*s < 1.15, *p*s > 0.288.

Since our gaze criteria for hold gaze trials only required participants to make at least one fixation at the to-be-attended location between cue onset and their behavioral response, it is possible that participants also made unnecessary fixations at the to-be-ignored location on some subset of accurate trials before redirecting their gaze to the correct location. These unnecessary, extra fixations could partly account for an increase in digit categorization RT and a decrease in accuracy for hold trials falling in high shift likelihood blocks if they were influenced by the expectation to shift attention. We therefore tested whether the degree to which participants fixated on the to-be-ignored location prior to making a response varied based on shift likelihood and sustained attention state with another repeated measures ANOVA with factors of sustained attention state (*in the zone*vs. *out of the zone*) and shift likelihood (low vs. high). For this analysis, we included all hold attention trials regardless of whether the participant also made an accurate fixation and/or the correct manual response, except for those trials in which the digits were replaced by an X due to an incorrect fixation appearing earlier in the trial (see Methods). The total number of trials per condition per participant ranged from 6 to 43. While the overall percentage of fixations at the to-be-ignored location did not differ according to states of sustained attention, *F*(1,74) = 1.17, *p* = 0.282, = 0.016, a significant main effect of shift likelihood revealed that participants were more likely to fixate at the incorrect location prior to responding if they were in a high shift likelihood context than in a low shift likelihood context, *F*(1,74) = 60.71, *p* < 0.001, = 0.451 (see Fig. 4D). Most interestingly, a significant interaction indicated that the difference in number of fixations at the to-be-ignored location was larger for *in the zone* contexts than for *out of the zone* contexts, suggesting that individuals were most likely to fail to suppress anticipatory fixations when CPT RT variability was low, *F*(1,74) = 6.07, *p* = 0.016, ${\widehat{\eta }}_{p}^{2}$= 0.076.”

Now reads:

“Overall, there was no evidence of a speed-accuracy tradeoff (see Fig. 4C). Participants were less accurate on shift gaze trials than on hold gaze trials, *F*(1,75) = 47.17, *p* < 0.001, ${\widehat{\eta }}_{p}^{2}$= 0.386, and when *out of the zon*e relative to periods *in the zone*, *F*(1,75) = 43.68, *p* < 0.001, ${\widehat{\eta }}_{p}^{2}$ = 0.368. No other main effects or interactions reached statistical significance. *F*s < 1.51 ps > 0.223.

Since our gaze criteria for hold gaze trials only required participants to make at least one fixation at the to-be-attended location between cue onset and their behavioral response, it is possible that participants also made unnecessary fixations at the to-be-ignored location on some subset of accurate trials before redirecting their gaze to the correct location. These unnecessary, extra fixations could partly account for an increase in digit categorization RT for hold trials falling in high shift likelihood blocks if they were influenced by the expectation to shift attention. We therefore tested whether the degree to which participants fixated on the to-be-ignored location prior to making a response varied based on shift likelihood and sustained attention state with another repeated measures ANOVA with factors of sustained attention state *(in the zone* vs. *out of the zone*) and shift likelihood (low vs. high). For this analysis, we included all hold attention trials with a manual response regardless of whether the participant also made an accurate fixation and/or the correct manual response, except for those trials in which the digits were replaced by an X due to an incorrect fixation appearing earlier in the trial or were excluded due to a stimulus presentation error (see Methods). The total number of trials per condition per participant ranged from 5 to 42. While the overall percentage of fixations at the to-be-ignored location did not differ according to states of sustained attention, *F*(1,75) = 0.07, *p* = 0.794, ${\widehat{\eta }}_{p}^{2}$ = 0.001, a significant main effect of shift likelihood revealed that participants were more likely to fixate at the incorrect location prior to responding if they were in a high shift likelihood context than in a low shift likelihood context, *F*(1,75) = 127.59, *F* < 0.001, ${\widehat{\eta }}_{p}^{2}$= 0.630 (see Fig. 4D). The interaction approached significance, *F*(1,75) = 3.73, p = 0.057, ${\widehat{\eta }}_{p}^{2}$= 0.047, indicating that the shift likelihood difference in number of fixations at the to-be-ignored location trended toward being larger for *in the zone* contexts than for *out of the zone*.”

In the caption for Figure 4:

**Figure 4**. The interaction of cue type, shift likelihood, and sustained attention state. A) Digit categorization RTs and B) saccade latencies as a function of trial type, shift likelihood context, and sustained attention state. A single participant is excluded from A-B due to missing data. C) Combined behavioral accuracies for manual responses and gaze position as a function of trial type, shift likelihood, and sustained attention state. D) The percentage of fixations made at the to-be-ignored location prior to manual response varied according to shift likelihood and sustained attention state. Error bars denote difference and correlation-corrected within-subject 95% confidence intervals.^27^ Correlation adjustment was carried out using the Cousineau-Morey approach.^28,29^

Now reads:

**Figure 4**. The interaction of cue type, shift likelihood, and sustained attention state. A) Digit categorization RTs and B) saccade latencies as a function of trial type, shift likelihood context, and sustained attention state. A single participant is excluded from A-B due to missing data. C) Combined behavioral accuracies for manual responses and gaze position as a function of trial type, shift likelihood, and sustained attention state. D) The percentage of fixations made at the to-be-ignored location prior to manual response varied according to shift likelihood. Error bars denote difference and correlation-corrected within-subject 95% confidence intervals.^27^ Correlation adjustment was carried out using the Cousineau-Morey approach.^28,29^

In the “Shift likelihood influences on sustained attention” subsection:

“We observed no significant difference in CPT RT variability across low shift likelihood (*M* = 0.13, *SD* = 0.03) and high shift likelihood (*M* = 0.13, *SD* = 0.03) contexts, *t*(74) = 0.90, *p* = 0.368, *d*_*z*_ = 0.104, indicating that participants were just as likely to fluctuate between high and low efficacy periods of sustained attention when in states of low and high shift readiness.

Relatedly, as stated above, our pre-cue VTC measure of sustained attention reflected CPT RT variability both before and after each cue presentation due to interpolation and smoothing, which does not allow for causal inferences. To determine the extent to which attentional orienting trials influenced ongoing changes in CPT RT variability, we averaged the non-interpolated and unsmoothed VTC values immediately after each digit categorization period and tested for effects of previous digit categorization outcomes (see Fig. 5). For each attentional orienting trial, we averaged up to three post-cue VTC values to estimate post-cue sustained attention, excluding CPT trials that were commission errors or response omissions since these trials would not have an associated non-interpolated RT. If there were no available VTC values to average, we excluded this orienting trial from the analysis (0.57% of all trials with a post-cue period). We subjected the post-cue VTC values following accurate trials to a repeated measures ANOVA with factors of trial type (shift vs. hold) and shift likelihood (low vs. high). Post-cue variability did not differ based on trial type, *F*(1,74) = 0.41, *p* = 0.526, ${\widehat{\eta }}_{p}^{2}$= 0.005, shift likelihood, *F*(1,74) = 0.02, *p* = 0.892, ${\widehat{\eta }}_{p}^{2}$< 0.001, nor their interaction,* F*(1,74) = 0.03, *p* = 0.853, ${\widehat{\eta }}_{p}^{2}$< 0.001 (see Fig. 5A). While there was no evidence that violations of shift expectations were associated with a post-cue change in sustained attention, an additional source of variability in CPT RTs may be post-error slowing following inaccurate digit categorization trials. We tested whether post-cue VTC differed based on whether participants made a correct response for the previous digit categorization using a paired samples *t*-test. Relative to the overall mean of each run, participants had more variable post-cue CPT RTs following a digit categorization error than after a correct response, *t*(74) = 6.86,* p* < 0.001, *d*_*z*_ = 0.792, reflecting a decrease in the efficacy of sustained attention (see Fig. 5B).”

Now reads:

“We observed no significant difference in CPT RT variability across low shift likelihood (*M* = 0.13, *SD* = 0.03) and high shift likelihood (*M* = 0.13, *SD* = 0.03) contexts, *t*(75) = 1.04, *p* = 0.304, *d*_*z*_ = 0.119, indicating that participants were just as likely to fluctuate between high and low efficacy periods of sustained attention when in states of low and high shift readiness.

Relatedly, as stated above, our pre-cue VTC measure of sustained attention reflected CPT RT variability both before and after each cue presentation due to interpolation and smoothing, which does not allow for causal inferences. To determine the extent to which attentional orienting trials influenced ongoing changes in CPT RT variability, we averaged the non-interpolated and unsmoothed VTC values immediately after each digit categorization period and tested for effects of previous digit categorization outcomes (see Fig. 5). For each attentional orienting trial, we averaged up to three post-cue VTC values to estimate post-cue sustained attention, excluding CPT trials that were commission errors or response omissions since these trials would not have an associated non-interpolated RT. If there were no available VTC values to average, we excluded this orienting trial from the analysis (0.59% of all trials with a post-cue period). We subjected the post-cue VTC values following accurate trials to a repeated measures ANOVA with factors of trial type (shift vs. hold) and shift likelihood (low vs. high). Post-cue variability did not differ based on trial type *F*(1,75) = 0.23, *p* = 0.631, ${\widehat{\eta }}_{p}^{2}$= 0.003, shift likelihood, *F*(1,75) < 0.01, *p* = 0.952, ${\widehat{\eta }}_{p}^{2}$< 0.001, nor their interaction, *F*(1,75) = 0.10, *p* = 0.755, ${\widehat{\eta }}_{p}^{2}$= 0.001 (see Fig. 5A). While there was no evidence that violations of shift expectations were associated with a post-cue change in sustained attention, an additional source of variability in CPT RTs may be post-error slowing following inaccurate digit categorization trials. We tested whether post-cue VTC differed based on whether participants made a correct response for the previous digit categorization using a paired samples t-test. Relative to the overall mean of each run, participants had more variable post-cue CPT RTs following a digit categorization error than after a correct response,* t*(75) = 6.84, *p* < 0.001, *d*_*z*_ = 0.784, reflecting a decrease in the efficacy of sustained attention (see Fig. 5B).”

Under the Discussion section the following sentence:

Furthermore, we manipulated the likelihood of gaze shifting over time, and individuals demonstrated robust statistical learning effects, such that the behavioral costs associated with gaze shifting in digit categorization RT, saccade latency, and accuracy were all larger when shifting was unlikely than when shifting was highly likely^16,18^.”

In the same paragraph:

“Specifically, there was a robust effect such that participants generally made more fixations at the to-be-ignored location when expecting to shift gaze than when expecting to hold. This effect was magnified when CPT RT variability was low, suggesting that participants most strongly anticipated, or were most driven by their predictions, when the efficacy of sustained attention was high.”

Now reads:

“Specifically, there was a robust effect such that participants generally made more fixations at the to-be-ignored location when expecting to shift gaze than when expecting to hold.”

In the Discussion section:

“Additionally, we observed evidence that high efficacy sustained attention was associated with an increase in the automaticity of saccade generation. Participants made more fixations at the to-be-ignored location when in high shift likelihood contexts than in low shift likelihood contexts, and this difference was amplified when in the zone. The increased likelihood to make an un-cued saccade indicates that when participants were *in the zone* and expecting to make a saccade, preparatory activity sometimes drove behavior more strongly than the unexpected hold stimulus. However, we did not explicitly count these trials as errors in the feedback that participants received. Although they received instructions to look wherever the color cue indicated, there was no penalty for exploring the opposing stream during the response window. Taken together, our results suggest that high efficacy sustained attention both enables the rapid disengagement from an unexpected shift cue and leads to a greater likelihood of anticipatory saccades when an overt shift is expected. An important topic for future study is whether the interaction we observed between shift readiness learning and states of sustained attention is specific to saccadic planning, or whether it applies more broadly to other unexpected stimuli.”

Now reads:

“Participants made more fixations at the to-be-ignored location when in high shift likelihood contexts than in low shift likelihood contexts. Furthermore, there was a trend that suggested this difference may be amplified when *in the zone*. However, we did not explicitly count trials with errant fixations as errors in the feedback that participants received. Although they received instructions to look wherever the color cue indicated, there was no penalty for exploring the opposing stream during the response window. Future research is needed to better understand the degree to which sustained attention may modulate learned anticipatory saccades.”

Furthermore, under the Methods section in subsection “Participants” the sentence:

“Twenty-four participants were excluded from our analyses for having overall behavioral accuracies (computed according to manual response and eye gaze criteria) below 70% (n = 13), not completing at least three runs of the experimental task (n = 2), an inability to track eye position (n = 7), or experiencing technical difficulties during participation (n = 2).”

“Twenty-three participants were excluded from our analyses for having overall behavioral accuracies (computed according to manual response and eye gaze criteria) below 70% (n = 12), not completing at least three runs of the experimental task (n = 2), an inability to track eye position (n = 7), or experiencing technical difficulties during participation (n = 2).

Under same section in subsection “Stimuli and procedure” the sentence:

“The stimuli consisted of 10 city and 10 mountain scenes, as well as 60 phase scrambled scenes, all of which have been used in previous studies^5,11,26^”

Now reads: “The stimuli consisted of 10 city and 10 mountain scenes, as well as 10 phase scrambled scenes, all of which have been used in previous studies^5,11,26^”.

In the same subsection a paragraph was added after the sentence “At the conclusion of the attention cue window, the colored borders and digits offset and the images inside each aperture returned to city and mountain scenes.” The paragraph reads:

“A stimulus presentation error affected the last trial of each run such that the trial ended prematurely during the last stage of image transition for some participants (n=29 among those retained in the final analysis) or the response was not recorded for the remaining participants (n=47 among those retained in the final analysis). We exclude any of these trials where no response was made or recorded because the failure to record a press could have resulted from the error. However, we include trials with an accurate press since any recorded press would have occurred prior to the premature trial end. Alternatively, excluding all final trials from each run produced the same pattern of results and did not change any conclusions.”

In the same subsection at the end of the second to last paragraph the following sentence:

“This procedure resulted in a reduction of 2.06% of all digit categorization trials across the entire sample, with 1–7 digit categorization trials excluded per participant (*M* = 3.84, *SD* = 1.55).”

Now reads:

“This procedure resulted in a reduction of 2.05% of all digit categorization trials across the entire sample, with 1–7 digit categorization trials excluded per participant (*M* = 3.82, *SD* = 1.56).”

Under the “Data analysis” subsection:

“Specifically, for hold gaze trials, we required that participants make at least one fixation at the to-be-attended location during the temporal window ranging from cue onset through the participant’s manual response, resulting in the exclusion of 4.03% of all potential hold trials from RT analyses. Similarly, for shift gaze trials, a trial was coded as accurate only if the participant made a saccade from the correct starting interest area to the other interest area in the temporal window between cue onset and manual response, resulting in the exclusion of 4.79% of all potential shift trials from RT and saccade latency analyses. In both cases, the interest areas matched the on-screen locations of the left and right streams. This definition of accuracy differed from that used to provide participants feedback, which was based on manual response accuracy alone (see above). Trials with an inaccurate response, no response, or that were marked incorrect due to a pre-cue fixation on the wrong side of the display consisted of 10.19% of all trials.”

Now reads:

“Specifically, for hold gaze trials, we required that participants make at least one fixation at the to-be-attended location during the temporal window ranging from cue onset through the participant’s manual response, resulting in the exclusion of 0.50% of all potential hold trials from RT analyses. Similarly, for shift gaze trials, a trial was coded as accurate only if the participant made a saccade from the correct starting interest area to the other interest area in the temporal window between cue onset and manual response, resulting in the exclusion of 4.27% of all potential shift trials from RT and saccade latency analyses. In both cases, the interest areas matched the on-screen locations of the left and right streams. This definition of accuracy differed from that used to provide participants feedback, which was based on manual response accuracy alone (see above). Trials with an inaccurate response, no response, or that were marked incorrect due to a pre-cue fixation on the wrong side of the display consisted of 8.10% of all trials.”

In the same section under subsection “Variance time course”:

“We assigned ambiguous presses between these points in time to trials according to an iterative algorithm (accounting for 28.86% of all non-scrambled image CPT trials). In these cases of ambiguity, we first assigned the press to whichever image did not already have an assigned press. If neither image had an assignment, we attributed the press to the adjacent image that was closest to 100% coherent (onsetting image > = 60% coherent, offsetting image < 60% coherent) unless one of the images was a mountain scene or the beginning or end of an attention cue window. In these cases, we assigned the press to the adjacent image that was not a mountain or scrambled image. For trials with multiple presses, we assigned RTs based on time, with the fastest RTs assigned first. On average, only 1.41% (*SD* = 1.47) of all presses per participant were left unassigned (range 0–10.55%) when excluding rare presses affected by a stimulus timing imperfection (see below), indicating that, overall, there was a strong correspondence between presses made and CPT trials.”

Now reads:

“We assigned ambiguous presses between these points in time to trials according to an iterative algorithm (accounting for 28.57% of all non-scrambled image CPT trials). In these cases of ambiguity, we first assigned the press to whichever image did not already have an assigned press. If neither image had an assignment, we attributed the press to the adjacent image that was closest to 100% coherent (onsetting image > = 60% coherent, offsetting image < 60% coherent) unless one of the images was a mountain scene or the beginning or end of an attention cue window. In these cases, we assigned the press to the adjacent image that was not a mountain or scrambled image. For trials with multiple presses, we assigned RTs based on time, with the fastest RTs assigned first. On average, only 1.39% (*SD* = 1.47) of all presses per participant were left unassigned (range 0–10.55%) when excluding rare presses affected by a stimulus timing imperfection (see below), indicating that, overall, there was a strong correspondence between presses made and CPT trials.”

At the end of the same subsection the following sentence was changed from:

“Exclusion of a single trial fitting this retention rule did not change any conclusions. In total, this procedure resulted in a reduction of fewer than one trial per participant on average (*M* = 0.19, *SD* = 0.59), and RTs for these trials were then interpolated as above.”

and now reads:

“Exclusion of a single trial fitting this retention rule did not change any conclusions. In total, this procedure resulted in a reduction of fewer than one trial per participant on average (*M* = 0.18, *SD* = 0.58), and RTs for these trials were then interpolated as above.”

In the subsection “Linear mixed-effects models” :

When the maximal model did not successfully converge, we again removed the correlations among random intercepts and slopes: RT∼1 + CueType + PupilSize + PupilSize^2^ + CueType ∗ PupilSize + CueType ∗ PupilSize^2^ + (1 + CueType + PupilSize + PupilSize^2^ + CueType ∗ PupilSize + CueType ∗ PupilSize^2^||participant).

Now reads:

“When the maximal model did not successfully converge, we again removed the correlations among random intercepts and slopes and then simplified again by removing the random slope associated with the interaction of cue type by the quadratic pupil size effect: RT∼1 + CueType + PupilSize + PupilSize^2^ + CueType ∗ PupilSize + CueType ∗ PupilSize^2^ + (1 + CueType + PupilSize + PupilSize^2^ + CueType*PupilSize ||participant).

Additionally in the same subsection:

“After the maximal model yielded a singular fit, we simplified the random effects structure by removing the estimated correlations among random intercepts and slopes and then again by removing random slopes for the quadratic effect: SaccadeLatency ∼ 1+PupilSize+PupilSize^2^+(1+PupilSize||participant).”

Now reads:

“After the maximal model yielded a singular fit, we simplified the random effects structure by removing the estimated correlations among random intercepts and slopes SaccadeLatency ∼1+PupilSize+PupilSize^2^+(1+PupilSize + PupilSize^2^||participant).”.

Furthermore, as result of the changes, Figures 2, 3, 4 and 5 have been adjusted to reflect the updated data. The original versions of the Figures alongside their legends appear below.

**Fig. 2:**
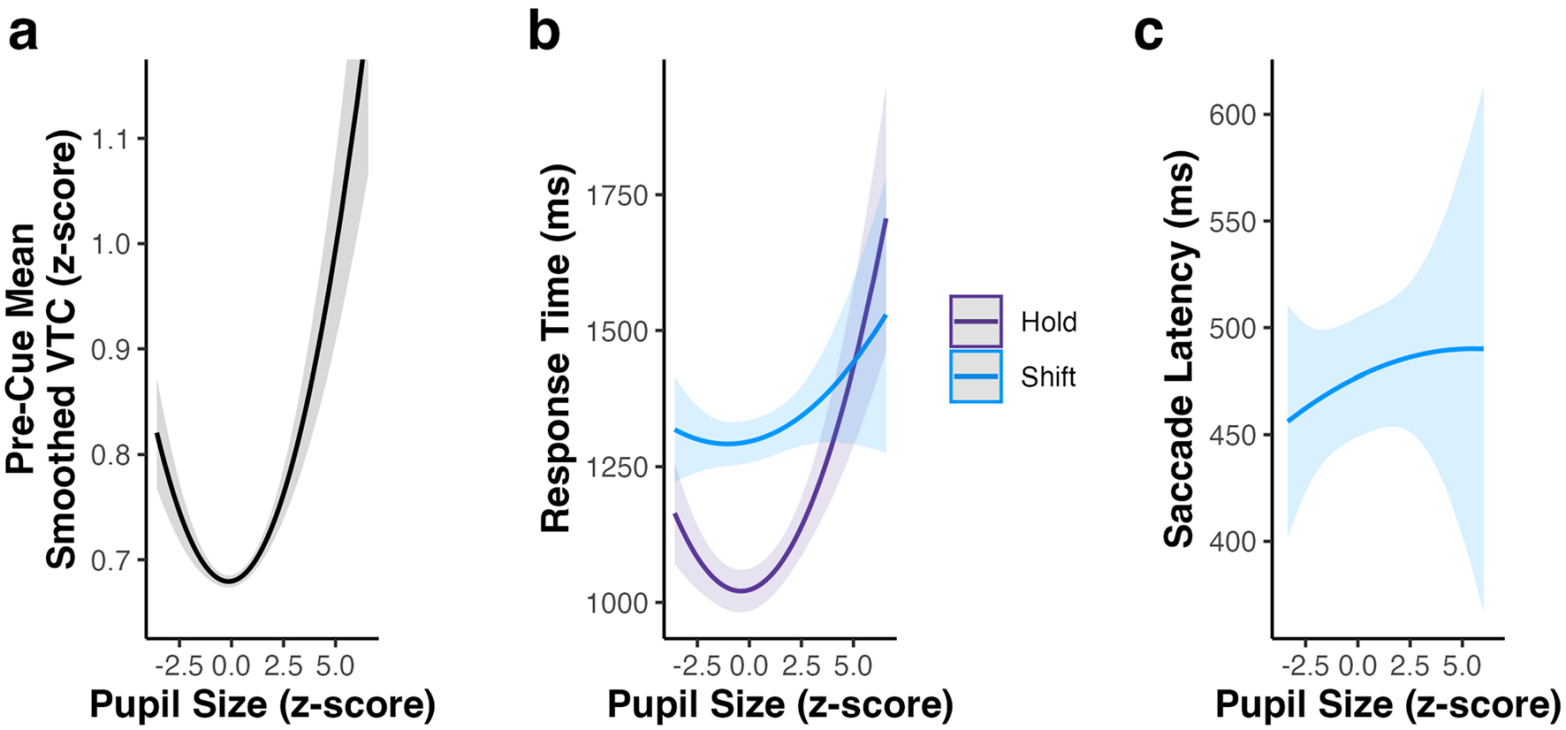
Marginal estimated fixed effects for (**a**) pre-cue RT, (**b**) digit categorization response time followinga hold or shift cue, and (**c**) saccade latency as a function of pretrial pupil size. Shaded regions denote 95%confidence intervals.

**Fig. 3:**
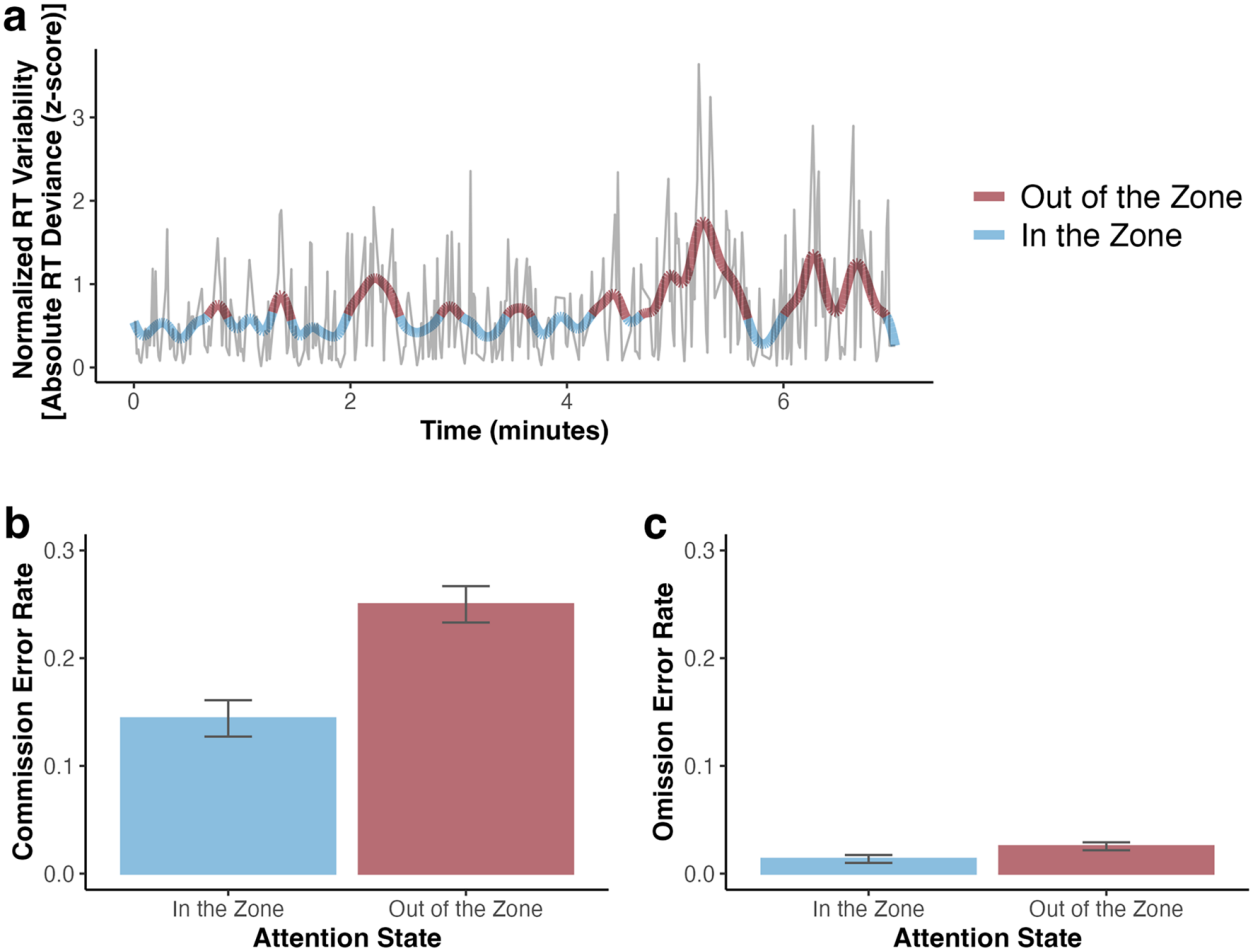
Definition of in the zone and out of the zone epochs. (**a**) Example VTC from a single run of oneparticipant. Raw RT variability is plotted in light gray. The thick line represents the smoothed time course withthe run divided into in the zone and out of the zone epochs according to a median split. (**b**) Commission and(c) omission error rates as a function of sustained attention state. Error bars denote difference and correlationcorrected within-subject 95% confidence intervals^27^. Correlation adjustment was carried out using theCousineau-Morey approach^28,29^.

**Fig. 4:**
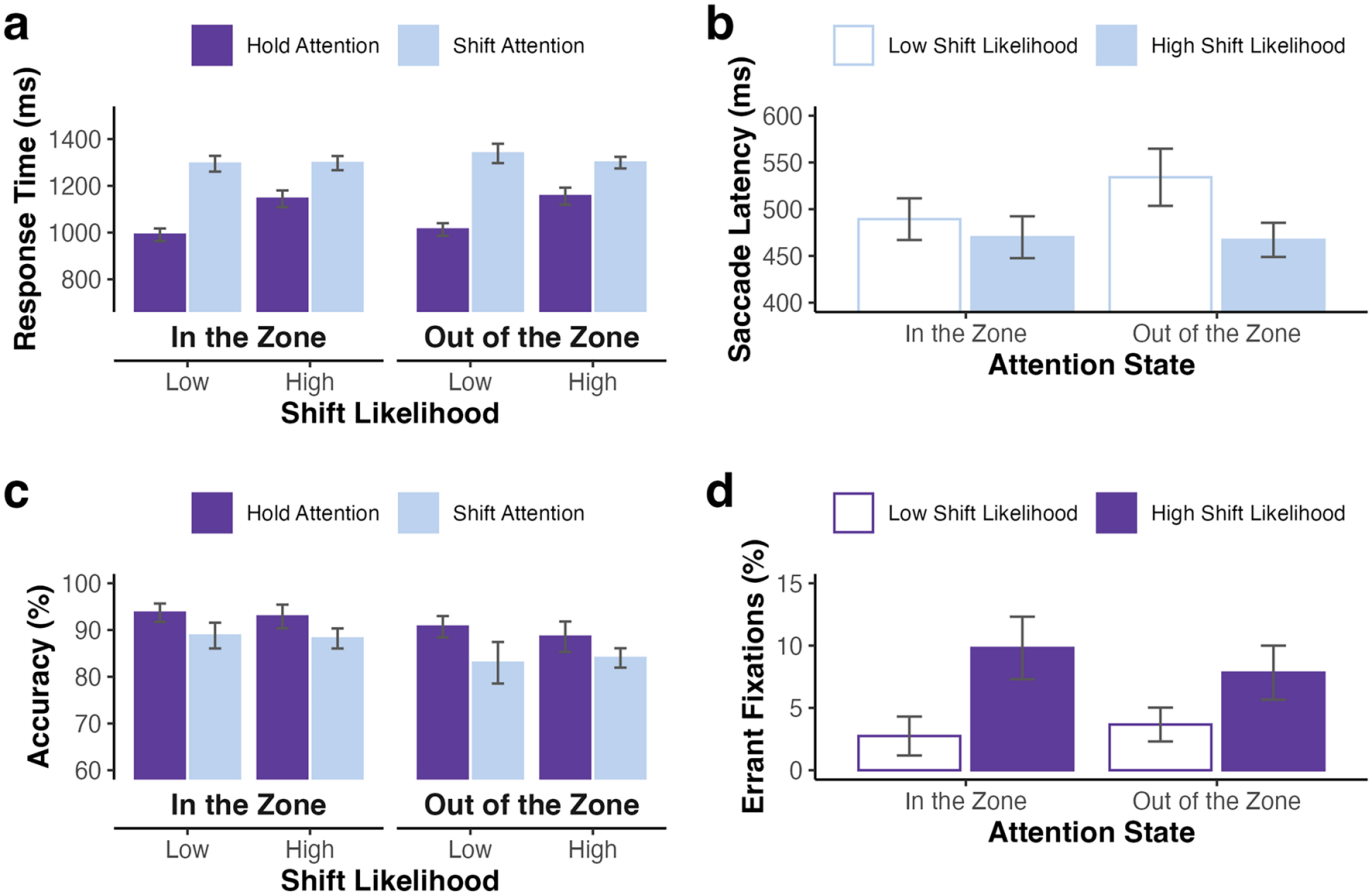
The interaction of cue type, shift likelihood, and sustained attention state. (**a**) Digit categorization RTsand (**b**) saccade latencies as a function of trial type, shift likelihood context, and sustained attention state. Asingle participant is excluded from A-B due to missing data. (**c**) Combined behavioral accuracies for manualresponses and gaze position as a function of trial type, shift likelihood, and sustained attention state. (**d**) Thepercentage of fixations made at the to-be-ignored location prior to manual response varied according to shiftlikelihood and sustained attention state. Error bars denote difference and correlation-corrected within-subject95% confidence intervals^27^. Correlation adjustment was carried out using the Cousineau-Morey approach^28,29^.

**Fig. 5:**
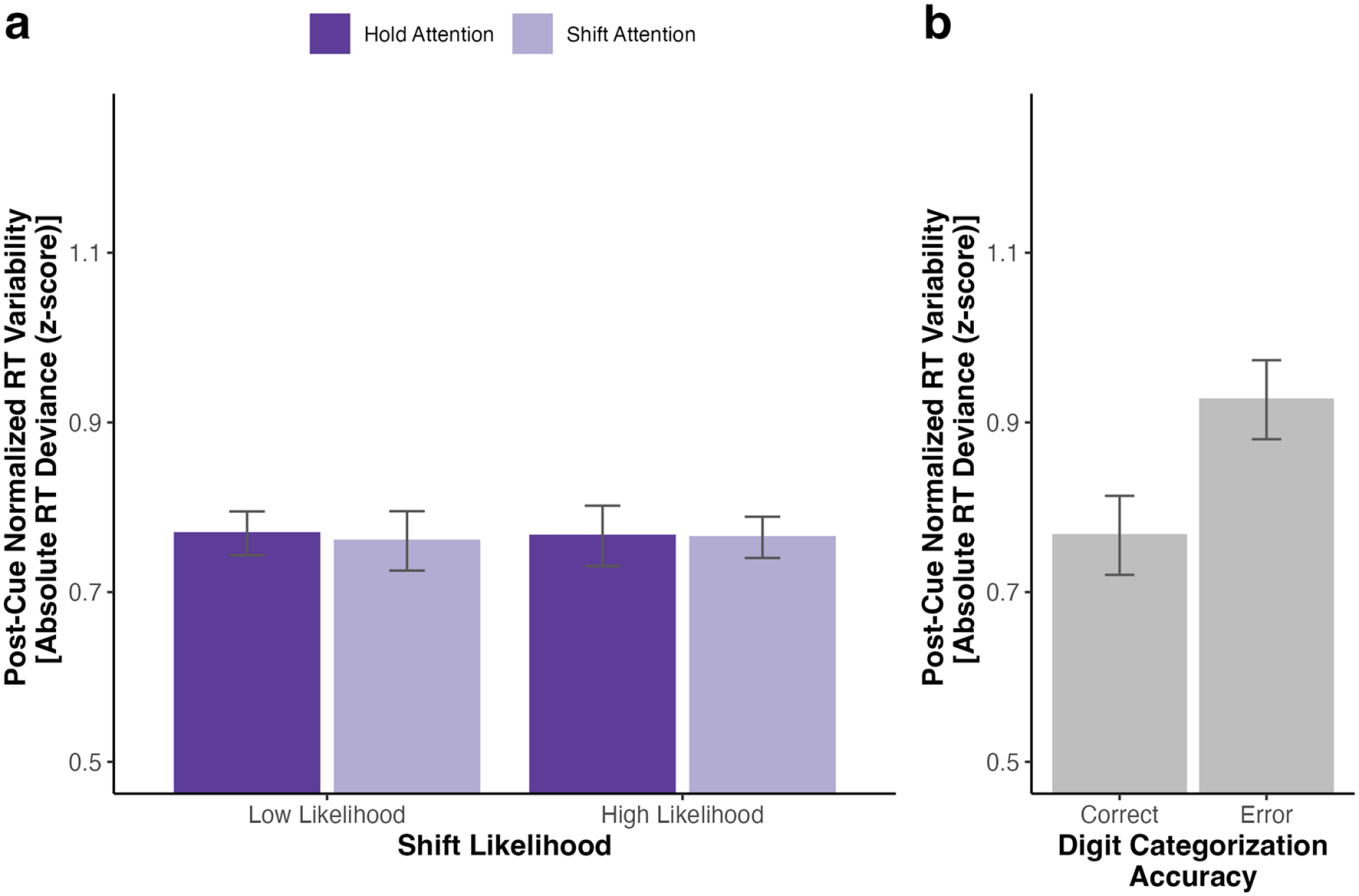
Post-attentional orienting cue non-interpolated and unsmoothed VTC as a function of (**a**) trialtype and shift likelihood and (**b**) previous digit categorization accuracy. Error bars denote difference andcorrelation-corrected within-subject 95% confidence intervals^27^. Correlation adjustment was carried out usingthe Cousineau-Morey approach^28,29^.

Lastly the Supplementary material has been replaced with an updated version that takes into account all the changes in the Article.

The original Article and Supplementary Material have been updated.

In addition to the correction, which will be linked to the published article, the following statement will be added to the Additional Information section of the article:

The original online version of this Article was revised: The original online version of this Article was revised: The original version of this Article contained errors. As a result, corrections have been made in the Results, Discussion, Methods, in Figures 2, 3, 4, and 5, and in the Supplementary Information file. Full information regarding the corrections made can be found in the correction for this Article.

## Supplementary Information

Supplementary Information.

