# Supplementary material for: Learned saccade readiness varies with fluctuations in sustained attention
Source: Sci Rep. 2025 Aug 23;15:31058. doi: 10.1038/s41598-025-14340-1 (PMC12374997; doi:10.1038/s41598-025-14340-1)
Supplement: Supplementary file 1 — Supplementary Material 1 [file 41598_2025_14340_MOESM1_ESM.docx]

**Supplemental Table S1.** Linear mixed-effects model fits when predicting pre-cue RT variability from pupil size.

|  | **Pre-Cue VTC** | | | |
| --- | --- | --- | --- | --- |
| *Predictors* | *Estimates* | *CI* | *p* | *df* |
| (Intercept) | 0.68 | 0.67 – 0.69 | **<0.001** | 107.31 |
| Pupil Size | 0.00 | -0.00 – 0.01 | 0.294 | 80.30 |
| Pupil Size^2^ | 0.01 | 0.01 – 0.02 | **<0.001** | 108.59 |
| **Random Effects** | | | | |
| σ^2^ | 0.06 | | | |
| τ_00_ _ID_ | 0.00 | | | |
| τ_11_ _ID.Pupil Size_ | 0.00 | | | |
| τ_11_ _ID.Pupil Size^2_ | 0.00 | | | |
| ICC | 0.00 | | | |
| N _ID_ | 76 | | | |
| Observations | 12128 | | | |
| Marginal R^2^ / Conditional R^2^ | 0.007 / 0.009 | | | |

*Note*. Marginal and conditional *R^2^* values are computed here with the sjPlot *R* package and may differ slightly from those reported in the main text. In the main text we used the *r2glmm* package for consistency with the semi-partial *R^2^* values reported.

**Supplemental Table S2.** Linear mixed-effects model fits when predicting digit parity categorization RTs from cue type and pupil size.

|  | **Response Time** | | | |
| --- | --- | --- | --- | --- |
| *Predictors* | *Estimates* | *CI* | *p* | *df* |
| (Intercept) | 1159.79 | 1121.95 – 1197.62 | **<0.001** | 75.85 |
| Pupil Size | 9.94 | 1.59 – 18.29 | **0.020** | 78.51 |
| Cue Type | -136.60 | -149.03 – -124.16 | **<0.001** | 90.91 |
| Pupil Size^2^ | 9.10 | 4.50 – 13.70 | **<0.001** | 66.51 |
| Pupil Size × Cue Type | 1.38 | -5.67 – 8.43 | 0.698 | 79.20 |
| Pupil Size^2^ × Cue Type | 5.01 | 1.24 – 8.78 | **0.009** | 2422.86 |
| **Random Effects** | | | | |
| σ^2^ | 98515.93 | | | |
| τ_00_ _ID_ | 26527.31 | | | |
| τ_11_ _ID.Pupil Size_ | 631.20 | | | |
| τ_11_ _ID.Cue Type_ | 2084.08 | | | |
| τ_11_ _ID.Pupil Size^2_ | 132.74 | | | |
| τ_11_ _ID.Cue Type x Pupil Size_ | 253.13 | | | |
| ICC | 0.21 | | | |
| N _ID_ | 76 | | | |
| Observations | 12128 | | | |
| Marginal R^2^ / Conditional R^2^ | 0.125 / 0.311 | | | |

*Note*. Marginal and conditional *R^2^* values are computed here with the sjPlot *R* package and may differ slightly from those reported in the main text. In the main text we used the *r2glmm* package for consistency with the semi-partial *R^2^* values reported.

**Supplemental Table S3.** Linear mixed-effects model fits when predicting saccade latency from pupil size.

|  | **Saccade Latency** | | | |
| --- | --- | --- | --- | --- |
| *Predictors* | *Estimates* | *CI* | *p* | *df* |
| (Intercept) | 477.00 | 448.56 – 505.43 | **<0.001** | 76.62 |
| Pupil Size | 4.76 | -1.89 – 11.42 | 0.158 | 79.43 |
| Pupil Size^2^ | -0.43 | -4.12 – 3.26 | 0.812 | 26.44 |
| **Random Effects** | | | | |
| σ^2^ | 44657.88 | | | |
| τ_00_ _ID_ | 14659.49 | | | |
| τ_11_ _ID.Pupil Size_ | 202.13 | | | |
| τ_11_ _ID.Pupil Size_^2^ | 2.72 | | | |
| ICC | 0.25 | | | |
| N _ID_ | 76 | | | |
| Observations | 5849 | | | |
| Marginal R^2^ / Conditional R^2^ | 0.000 / 0.247 | | | |

*Note*. Marginal and conditional *R^2^* values are computed here with the sjPlot *R* package and may differ slightly from those reported in the main text. In the main text we used the *r2glmm* package for consistency with the semi-partial *R^2^* values reported.

**Supplemental Table S4.** Linear mixed-effects model fits when predicting digit parity categorization RTs from cue type, shift likelihood, and sustained attention state.

|  | **Response Time** | | | |
| --- | --- | --- | --- | --- |
| *Predictors* | *Estimates* | *CI* | *p* | *df* |
| (Intercept) | 1190.06 | 1152.18 – 1227.94 | **<0.001** | 76.15 |
| Cue Type | -113.74 | -125.63 – -101.85 | **<0.001** | 83.71 |
| Shift Likelihood | 35.73 | 27.41 – 44.05 | **<0.001** | 82.75 |
| Sustained Attention | -9.81 | -17.80 – -1.83 | **0.016** | 111.81 |
| Cue Type × Shift Likelihood | 41.09 | 34.58 – 47.59 | **<0.001** | 11977.90 |
| Cue Type × Sustained Attention | 1.72 | -4.81 – 8.25 | 0.606 | 11985.07 |
| Shift Likelihood × Sustained Attention | 6.26 | -0.28 – 12.81 | 0.061 | 12007.31 |
| Cue Type × Shift Likelihood × Sustained Attention | -2.50 | -9.05 – 4.05 | 0.454 | 12004.23 |
| **Random Effects** | | | | |
| σ^2^ | 97160.27 | | | |
| τ_00_ _ID_ | 26648.57 | | | |
| τ_11_ _ID.Cue Type_ | 1870.80 | | | |
| τ_11_ _ID.Shift Likelihood_ | 488.11 | | | |
| τ_11_ _ID.Sustained Attention_ | 385.13 | | | |
| ICC | 0.22 | | | |
| N _ID_ | 76 | | | |
| Observations | 12163 | | | |
| Marginal R^2^ / Conditional R^2^ | 0.137 / 0.323 | | | |

*Note*. Marginal and conditional *R^2^* values are computed here with the sjPlot *R* package and may differ slightly from those reported in the main text. In the main text we used the *r2glmm* package for consistency with the semi-partial *R^2^* values reported.

**Supplemental Table S5.** Linear mixed-effects model fits when predicting saccade latencies from shift likelihood, and sustained attention state.

|  | **Saccade Latency** | | | |
| --- | --- | --- | --- | --- |
| *Predictors* | *Estimates* | *CI* | *p* | *df* |
| (Intercept) | 486.50 | 458.61 – 514.40 | **<0.001** | 75.24 |
| Shift Likelihood | -18.34 | -27.48 – -9.19 | **<0.001** | 75.90 |
| Sustained Attention | -9.37 | -16.27 – -2.48 | **0.008** | 105.94 |
| Shift Likelihood × Sustained Attention | 9.74 | 2.58 – 16.90 | **0.008** | 102.77 |
| **Random Effects** | | | | |
| σ^2^ | 43743.89 | | | |
| τ_00_ _ID_ | 14088.00 | | | |
| τ_11_ _ID.Shift Likelihood_ | 794.15 | | | |
| τ_11_ _ID.Sustained Attention_ | 101.15 | | | |
| τ_11_ _ID.Shift Likelihood × Sustained Attention_ | 169.38 | | | |
| ICC | 0.24 | | | |
| N _ID_ | 76 | | | |
| Observations | 5860 | | | |
| Marginal R^2^ / Conditional R^2^ | 0.006 / 0.248 | | | |

*Note*. Marginal and conditional *R^2^* values are computed here with the sjPlot *R* package and may differ slightly from those reported in the main text. In the main text we used the *r2glmm* package for consistency with the semi-partial *R^2^* values reported.
